# Comparative proteomic investigation of multiple methicillin-resistant *Staphylococcus aureus* strains generated through adaptive laboratory evolution

**DOI:** 10.1016/j.isci.2021.102950

**Published:** 2021-08-06

**Authors:** Jordy Evan Sulaiman, Lexin Long, Long Wu, Pei-Yuan Qian, Henry Lam

**Affiliations:** 1 Department of Chemical and Biological Engineering, The Hong Kong University of Science & Technology, Clear Water Bay, Kowloon, Hong Kong; 2 Department of Ocean Science and Hong Kong Branch of Southern Marine Science and Engineering Guangdong Laboratory, Guangzhou, The Hong Kong University of Science & Technology, Clear Water Bay, Kowloon, Hong Kong

**Keywords:** Microbiology, Evolutionary biology, Proteomics

## Abstract

Recent discoveries indicate that tolerance and resistance could rapidly evolve in bacterial populations under intermittent antibiotic treatment. In the present study, we applied antibiotic combinations in laboratory experiments to generate novel methicillin-resistant *Staphylococcus aureus* strains with distinct phenotypes (tolerance, resistance, and suppressed tolerance), and compared their proteome profiles to uncover the adaptation mechanisms. While the tolerant strains have very different proteomes than the susceptible ancestral strain, the resistant strain largely resembles the ancestral in terms of their proteomes. Our proteomics data and other assays support the connection between the detected mutations to the observed phenotypes, confirming the general understanding of tolerance and resistance mechanisms. While resistance directly counteracts the action mechanism of the antibiotic, tolerance involves complex substantial changes in the cells’ biological process to achieve survival advantages. Overall, this study provides insights into the existence of diverse evolutionary pathways for tolerance and resistance development under different treatment scenarios.

## Highlights

• Laboratory evolution led to strains with distinct tolerance/resistance phenotypes
• DAP/RIF combination applied to a DAP-tolerant strain increases its susceptibility
• Protein network of DAP-tolerant strain is more perturbed than the resistant strain
• Novel tolerance-related proteins revealed by proteomics

## Graphical abstract

**Figure:**
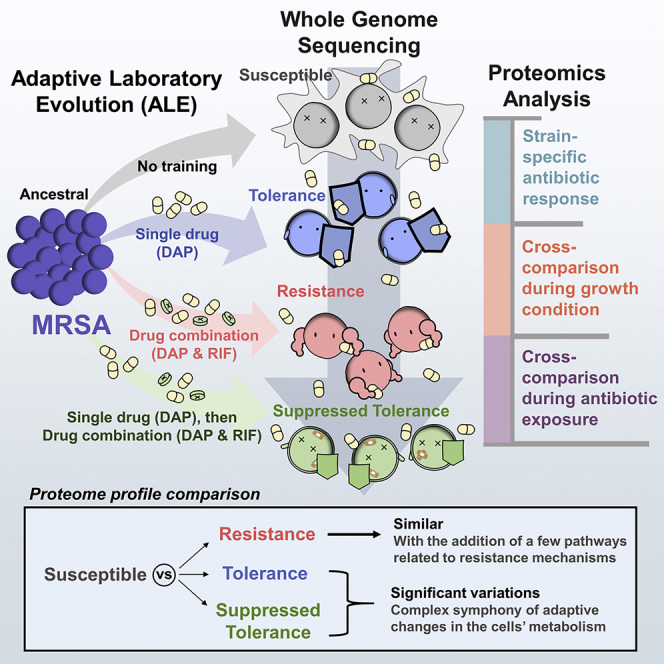

## Introduction

*Staphylococcus aureus* is a gram-positive bacterium that has long been recognized as one of the most important human pathogens (Lowy, 1998). Methicillin-resistant *S*. *aureus* (MRSA) strains, which are resistant to antibiotics including methicillin and other commonly used antibiotics, has spread widely and become endemic in most countries worldwide and is a leading cause of hospital-associated infections in the developed countries (Jarvis et al., 2007; Klein et al., 2007). Although β-lactams are usually preferred to treat methicillin-susceptible *S*. *aureus*, it is unable to kill MRSA. Other antimicrobials including fluoroquinolones and third-generation cephalosporins are also ineffective against most MRSA strains (Maranan et al., 1997), and only a few glycopeptide and lipopeptide antibiotics such as vancomycin (VAN) and daptomycin (DAP) are approved for MRSA treatment in clinics. Although VAN is commonly chosen as the first-line drug for treating patients suspected of MRSA infections (Fridkin et al., 1999), it has a lot of shortcomings including slow bacterial killing and poor tissue and intracellular penetration (Humphries et al., 2013). Moreover, with the increasing use of VAN, there have been multiple reports of MRSA isolates with elevated minimum inhibitory concentrations (MICs) toward VAN (>1 mg/L). Therefore, the use of DAP, another US Food and Drug Administration-approved lipopeptide antibiotic for MRSA treatments, is increasingly recognized, especially for MRSA strains with elevated VAN MICs (Murray et al., 2013). In addition, clinical treatments tend to involve several drugs, especially for patients with severe infections. For instance, rifampin (RIF), a bactericidal antibiotic that has an excellent penetration ability and is effective against cells in all growth phases, is regarded as an ideal adjuvant agent. Despite the excellent activity of RIF toward MRSA, it cannot be used alone due to the rapid emergence of resistance (Hughes and Brandis, 2013). That is why the combination of DAP and RIF has been repetitively used to treat MRSA (John et al., 2009; Saleh-Mghir et al., 2011).

Recently, several groups have shown that tolerance and resistance evolved rapidly under frequent, cyclic antibiotic treatment (Fridman et al., 2014; Mechler et al., 2015; Van den Bergh et al., 2016; Khare and Tavazoie, 2020; Sulaiman and Lam, 2020a; Sulaiman and Lam, 2020b). Tolerance and resistance are two different mechanisms by which bacteria survive antibiotic assault (Lewis, 2007; Brauner et al., 2016; Sulaiman and Lam, 2019). Unlike resistance mutations that compromise the effectiveness of the drug and allow the bacteria to grow at a higher antibiotic concentration (Blair et al., 2015), tolerance mutations allow the population to survive lethal antibiotic treatment for a prolonged duration (Brauner et al., 2017; Sulaiman and Lam, 2020b). While resistance is characterized through an elevation in the MIC, a tolerant population is marked by the higher minimum duration for killing (MDK) as they have no change in the MIC compared to the susceptible population (Brauner et al., 2016). Most of the previous studies that applied such *in vitro* evolution experiments to study tolerance and resistance only used a single drug (Sulaiman and Lam, 2021), and the evolutionary trajectory of tolerance and resistance development under drug combinations remains unexplored. A recent study by Liu et al. showed that such evolution of tolerance and resistance not only occurs *in vitro*, but also in clinical patients with MRSA blood infections that received drug combination treatment with DAP and RIF (Liu et al., 2020). They observed that once the cells have gained tolerance to DAP, the subsequent use of the drug combination increases the chance for RIF resistance to emerge. In other words, resistance development is promoted under drug combination if the bacteria have previously evolved tolerance. However, since this finding was obtained by isolating bacterial strains from a clinical patient over the treatment period, the dynamics of tolerance evolution might be affected by other factors such as host-microbe interactions. Therefore, studying the evolution of tolerance and resistance on the MRSA pathogen in a well-controlled setting using adaptive laboratory evolution (ALE) experiments should be of particular interest.

In this study, we monitored the development of tolerance and resistance in MRSA by treating them with either DAP alone, or DAP combined with RIF, in a cyclic manner. After two weeks of daily antibiotic treatments, the evolved populations from different treatment schemes exhibit distinct tolerance and resistance phenotypes. By whole-genome sequencing, we uncovered the genetic basis of the tolerance and resistance phenotypes in the evolved strains in the form of single-point mutations. Then, we compared the proteome profile of the evolved strains with different phenotypes in the absence and presence of an antibiotic. To our knowledge, there has not been any study that provides a comprehensive proteome comparison between resistant and tolerant bacterial populations, let alone ones that evolve from the same ancestor and differ minimally in genotypes. Therefore, our data should provide unprecedented insights into these two distinct adaptation mechanisms of MRSA against antibiotic assault.

## Results

### MRSA with distinct phenotypes evolved from different antibiotic treatment schemes

**Figure 1:**
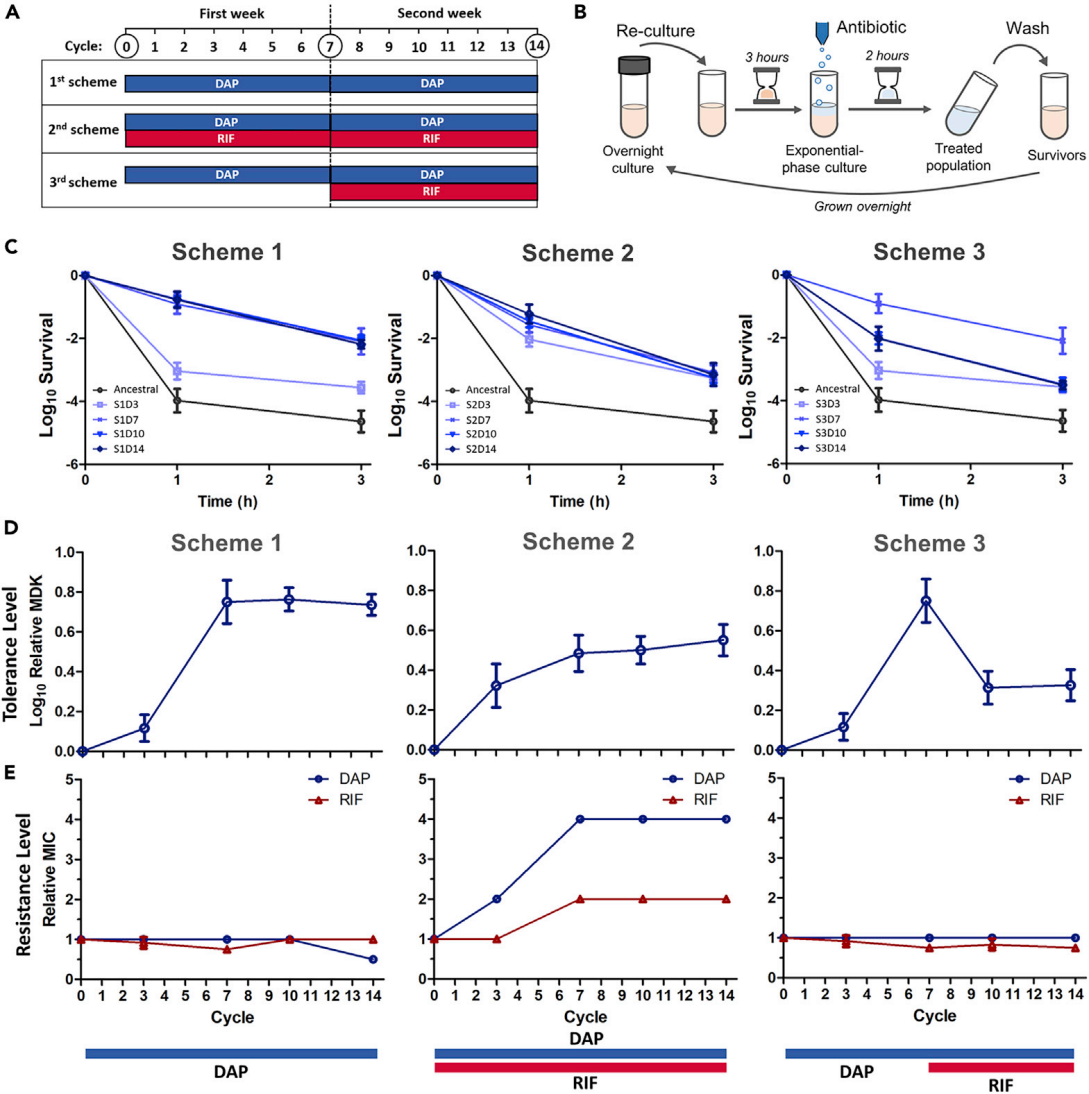
Evolution of tolerance and resistance on MRSA under different treatment schemes (A) Timeline of the evolution experiment on MRSA. In the first scheme, the culture was treated with DAP (10 μg/mL) for 2 h intermittently for two weeks. In the second scheme, the culture was treated with DAP (10 μg/mL) and RIF (1 μg/mL) combination for 2 h intermittently for two weeks. In the third scheme, the culture was treated with DAP (10 μg/mL) for 2 h intermittently for one week, and RIF (1 μg/mL) was added to the treatment regime in the second week. Population names are based on scheme (S#) and day after treatment (D#). (B) Schematic of the evolution experiment protocol. (C) Time-kill curve of ancestral MRSA and evolved populations after 3, 7, 10 and 14 days of treatment under scheme 1 (**left**), scheme 2 (**middle**) or scheme 3 (**right**) with DAP (10 μg/mL) (mean ±s.d., n = 5). (D and E) Relative MDK_99_ (minimum duration for killing 99% of the population) for DAP (mean ±s.d., n = 5) (D), and MIC for DAP and RIF (mean ±s.d., n = 3) (E) of the population before and after 3, 7, 10 and 14 days of treatment under scheme 1, scheme 2 or scheme 3. The colored bars below the graph indicate the antibiotic treatment regime.

**Figure 2:**
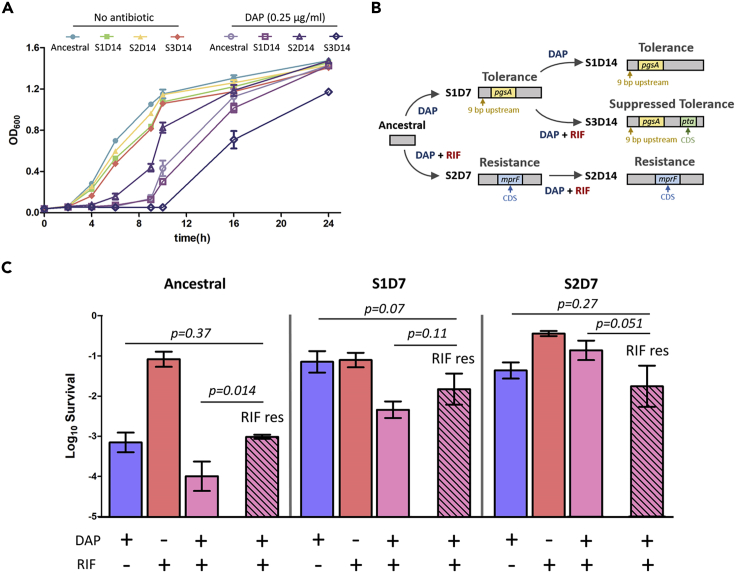
Characterization of the evolved strains (A) Growth profile of ancestral, S1D14, S2D14, and S3D14 populations in the absence and presence of DAP (0.25 μg/mL) (mean ±s.d., n = 3). (B) Schematic of single point mutations identified in the strains evolved from scheme 1, 2 and 3. CDS, coding sequence.c, Survival of the ancestral population, S1D7 and S2D7, and their RIF-resistant derivative (*rpoB*H481Y) (patterned fill) after 1 h of antibiotic treatments [DAP (10 μg/mL) and RIF (1 μg/mL)] (mean ±s.d., n = 3). p values for the pairwise comparison were estimated with two-tailed Student’s *t*-test with unequal variances of the log-transformed values.

We followed the evolution of methicillin-resistant *S*. *aureus* under different drug treatment schemes. The timeline and the protocol for the evolution experiment are shown in Figures 1A and 1B, where the bacterial culture was treated with DAP alone (scheme 1) or with DAP and RIF (DAP/RIF) combination (scheme 2) for two weeks. In addition, inspired by the study of Liu et al. (Liu et al., 2020), we also investigated an additional scenario where DAP was initially given in the first week, and then RIF was added to the treatment regime during the second week (scheme 3). This type of treatment scheme is sometimes undertaken in the clinic when the initial single-drug treatment does not yield the desired outcome, and a second drug is added. Under both single-drug treatment with DAP and DAP/RIF combination treatment, we observed that DAP tolerance quickly established in the populations, as shown by the reduced killing rate toward DAP after a few days of intermittent antibiotic treatment (Figure 1C**; left, middle**). After two weeks of evolution experiment, the strain from intermittent DAP treatment (S1D14) and the strain from intermittent DAP/RIF combination (S2D14) showed a much higher survival under DAP treatment than the ancestral strain (FigureS1). The increased tolerance in both strains was further confirmed by measuring their MDK_99_ values (Figure 1D). However, while S1D14 was merely tolerant to DAP (no MIC increase), S2D14 also showed a 4-fold increased MIC toward DAP, surpassing the EUCAST breakpoint for DAP resistance (Figure 1E**; middle,** disc diffusion assay is shown in FigureS2). Interestingly, when the population evolved from a week of DAP treatment was subsequently treated with drug combination (scheme 3), there was a significant drop in survival (Figure 1C**; right**), suggesting that drug combination increased the susceptibility (over 3-fold decrease in MDK_99_ and 2-fold decrease in MIC) of the previously DAP-tolerant population (Figures 1D and 1E**; right**).

Growth measurements showed that while the ancestral and S2D14 populations had similar growth profile, S1D14 and S3D14 had lower growth rates compared to the ancestral strain (Figure 2A), which is consistent with findings in previous studies (Van den Bergh et al., 2016; Sulaiman and Lam, 2020a). At a subinhibitory dose of 0.25 μg/mL of DAP, ancestral and S1D14 showed similar growth profile, while S2D14 grew faster due to their increased MIC toward DAP (4 μg/mL), and S3D14 grew slower due to their decreased MIC toward DAP (0.5 μg/mL).

### Single-point mutations on the evolved MRSA strains

To unearth the underlying adaptation mechanism for the different phenotypes, we proceeded to isolate colonies from each of the populations and sequenced their genome (Figure 2B and TablesS1 and S2). The evolved resistant population (S2D14) harbored a mutation in the *mprF* gene (T345A), which codes for phosphatidylglycerollysyltransferase. The DAP-tolerant strains S1D14 and S3D14 harbored a mutation in a non-coding region, 9 base-pairs upstream the *pgsA* gene, which codes for CDP-diacylglycerol-glycerol-3-phosphate-3-phosphatidyltransferase. Unlike the previously reported DAP-resistance through mutations in the *pgsA* gene (Peleg et al., 2012; Hines et al., 2017), our mutation does not occur in the gene, but just before the coding sequence (CDS), and we observed a tolerance phenotype instead of resistance. Interestingly, the S3D14 population, which has suppressed DAP tolerance after DAP/RIF combination treatment in the second week, retained the mutation upstream of *pgsA* but acquired an additional mutation in the CDS of the *pta* gene, coding for phosphate acetyltransferase, which is involved in the synthesis of acetyl phosphate from acetyl-CoA.

### RIF resistance is not allowed to evolve in the DAP-tolerant and resistant strains under drug combination treatment

Next, we were interested in knowing why RIF resistance is not established in the populations that were treated with the DAP/RIF combination. The slight increase in MIC of RIF after a week of DAP/RIF combination treatment (Figure 1E**; middle**) is not considered RIF resistance, which is commonly indicated by a thousand-fold increase in MIC (such as those that have mutations in the *rpoB* gene (Agha et al., 2020; Panchal et al., 2020)). To check the behavior of RIF-resistant mutants from different evolved strains under antibiotic treatment, we isolated the RIF-resistant mutants from the ancestral, S1D7, and S2D7 background by plating the cells on an agar plate containing RIF. The MIC of the RIF-resistant derivatives (which harbor H481Y mutation in the *rpoB* gene, Table S1) toward RIF is above 100 μg/mL (>10,000× MIC of the parental strains). This suggested that rare RIF-resistant mutants do occur in the three populations, but only in some cases can they establish in the whole population by out-competing their non-resistant counterparts.

We measured the survival of the ancestral and the evolved strains after 7 days of treatment toward each drug alone and in combination and found that DAP/RIF combination kills the susceptible ancestral population more effectively by a factor of 10 compared to DAP alone (Figure 2C**; left**). Under DAP/RIF combination treatment, the RIF-resistant derivative from the ancestral background had higher survival than the RIF-susceptible ancestral strain (p = 0.014). Therefore, RIF resistance is allowed to evolve in the ancestral population under the DAP/RIF combination treatment. This agreed with theoretical models which show that drug pairs with positive interaction (such as DAP and RIF), preferred clinically for their immediate efficacy, may eventually promote the evolution of resistance (Michel et al., 2008; Saputra et al., 2018). This may explain the slight increase in MIC toward RIF after several cycles of DAP/RIF combination treatment (Figure 1E**; middle**).

**Figure 3:**
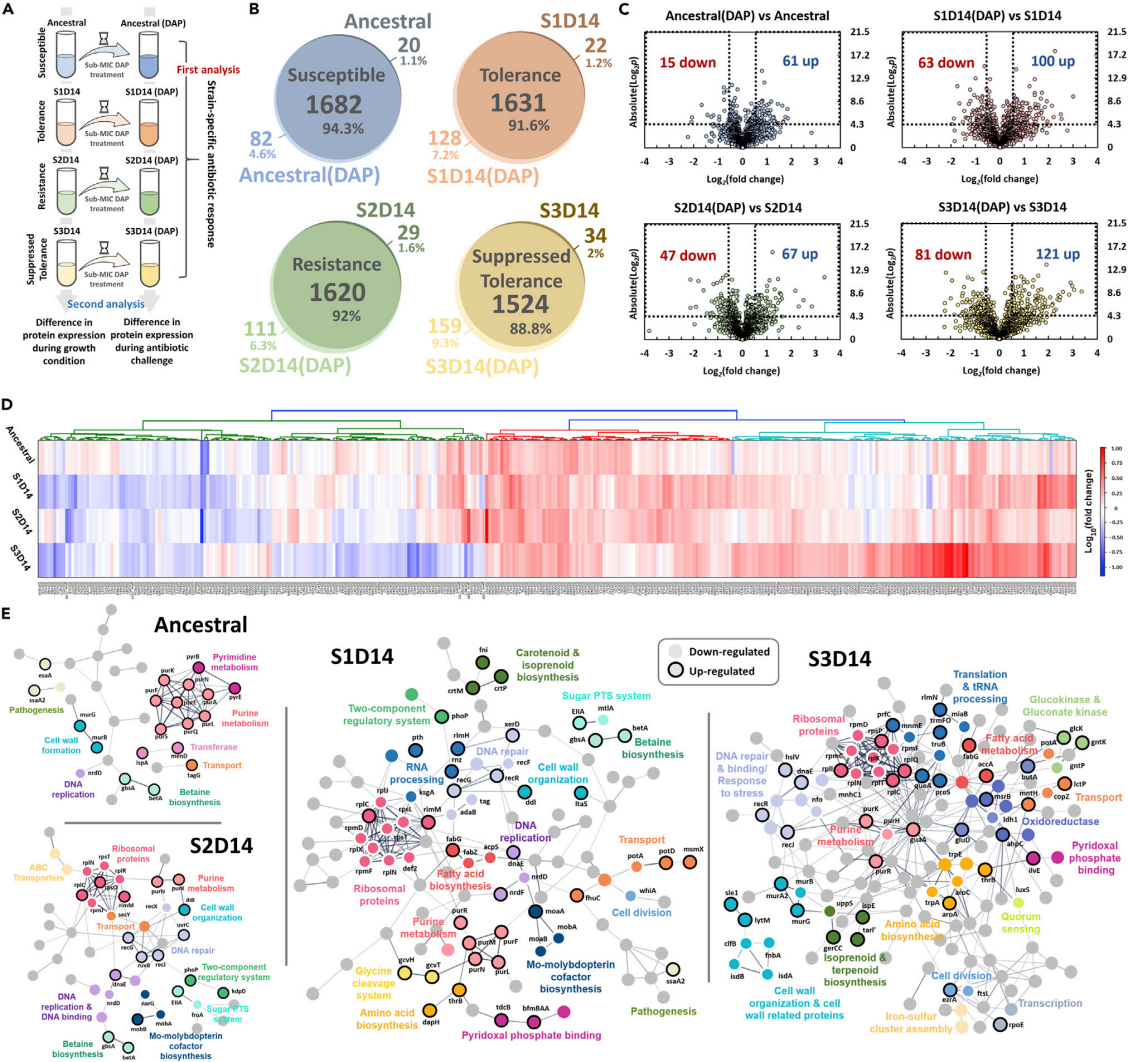
Proteomic response of ancestral, S1D14, S2D14 and S3D14 strains after DAP treatment (A) Schematic of proteomics data analysis strategy. First, we compared the proteome profile of each strain after DAP treatment to those before treatment (as controls) to obtain the strain-specific antibiotic response. Then, we also compared the proteome profile of the evolved strains with the ancestral as our control, in the presence and absence of DAP, to pinpoint the commonalities and differences in protein expression profiles between the evolved strains and the ancestral strain, under both normal growth condition and during antibiotic exposure. (B) Venn diagrams for proteome comparison of ancestral, S1D14, S2D14, and S3D14 populations before and after 1 h of DAP treatment (0.25 μg/mL). (C) Volcano plots for ancestral, S1D14, S2D14, and S3D14 populations after 1 h of DAP treatment compared with those before treatment. Differentially expressed proteins (DEPs) are defined to be those with pvalues below 0.05, and absolute fold change greater than 1.5, corresponding to the rectangular regions. Left rectangular regions are the down-regulated proteins and right rectangular regions are the up-regulated proteins. (D)Heatmap of the DEPs across the ancestral, S1D14, S2D14, and S3D14 populations under DAP treatment compared to the untreated populations. Hierarchical clustering was performed using Euclidean distance and a ward linkage model. (E) Protein-protein interaction network of the DEPs of ancestral and evolved populations when treated with DAP compared to the untreated populations, as predicted by STRING v11.0. The lines represent protein interaction (thicker lines mean higher confidence), and the dots in different colors represent different protein functions. Nodes with black outlines are up-regulated proteins, and nodes with white outlines are down-regulated proteins. Uncharacterized proteins are not annotated and nodes without function enrichment are colored gray.

However, after a week of intermittent DAP/RIF treatment (scheme 2), the drug combination became less effective by over three orders of magnitude (Figure 2C**; right**). Unlike the ancestral population, now the survival of the RIF-resistant derivative from the S2D7 background was no longer higher than the RIF-susceptible S2D7 strain. Therefore, under continued DAP/RIF combination treatment, the MIC toward RIF would no longer be increased (as shown by the constant value of MIC in the second week, Figure 1E**; middle**). Interestingly, this trend was not only observed in the resistant strain, but also in the DAP-tolerant strain (S1D7). When this strain was treated with DAP/RIF in the second week, the RIF-resistant derivative from the S1D7 background had similar survival compared to the RIF-susceptible S1D7 strain (p = 0.11) (Figure 2C**; middle**). Therefore, it is reasonable that RIF resistance will not emerge in S1D7 under drug combination treatment. To verify this explanation, we performed competition experiments where a small population of RIF-resistant mutants was mixed with their parental strains, and then treated with DAP/RIF combination. While the RIF-resistant mutants survived if they came from the ancestral background, we observed a significant drop in the number of RIF-resistant mutants in the two evolved strains (FigureS3).

### Proteomic response of the resistant strain is similar to the ancestral upon DAP treatment, while the tolerant strains have a more complicated response

**Figure 4:**
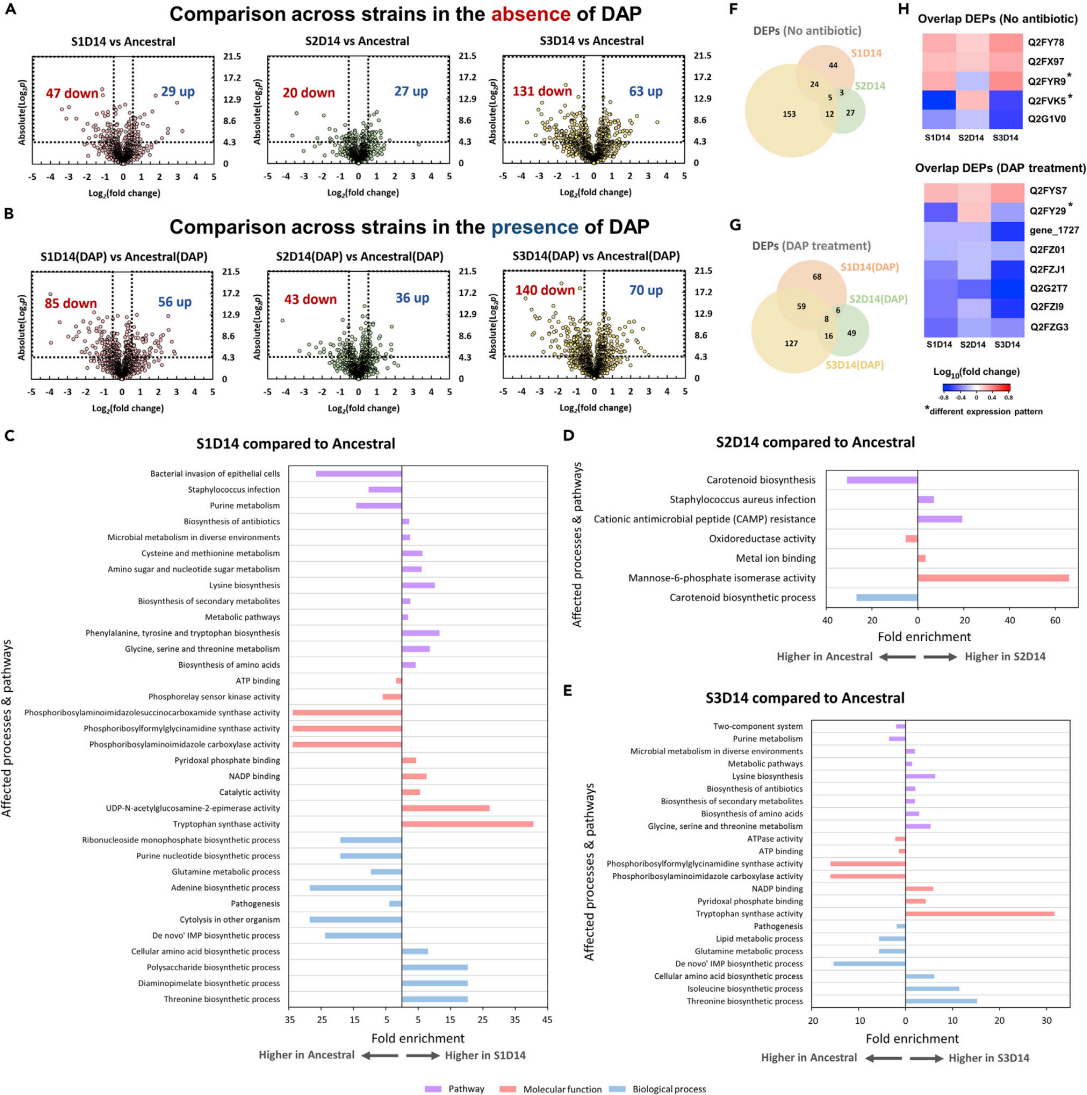
Proteome profile comparison between the evolved strains (S1D14, S2D14, and S3D14) and the ancestral strain (A and B) Volcano plots for S1D14, S2D14, and S3D14 populations compared to the ancestral in the absence of antibiotic (A) and after 1 h of DAP treatment (B). Differentially expressed proteins (DEPs) are defined to be those with pvalues below 0.05, and absolute fold change greater than 1.5, corresponding to the rectangular regions. Left rectangular regions are the down-regulated proteins (expression higher in the ancestral strain) and right rectangular regions are the up-regulated proteins (expression higher in the evolved strain). (C–G) Gene Ontology (GO) analysis and pathway enrichment study (KEGG) by DAVID of the DEPs of S1D14 compared to the ancestral (C), S2D14 compared to the ancestral (D) and S3D14 compared to the ancestral (E) after DAP treatment. Fold enrichment is defined as the ratio of the proportion of the input information to the background information. f, g, Venn diagrams of the DEPs comparison of S1D14, S2D14, and S3D14 populations (compared to ancestral) in the absence of antibiotic (F) and after 1 h of DAP treatment (G). (H) Overlapped differentially expressed proteins among the evolved strains in the absence (top) and presence (bottom) of daptomycin.

**Figure 5:**
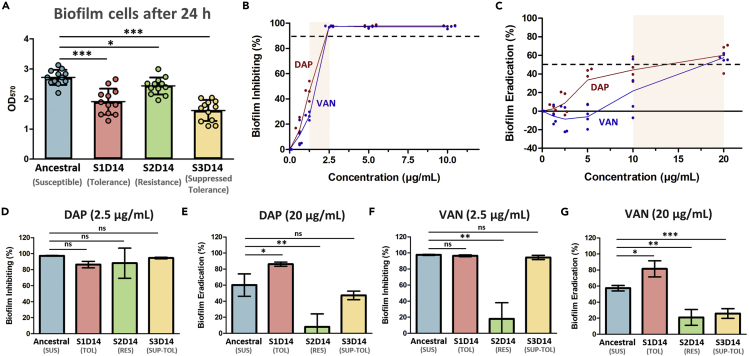
Biofilm assay of the ancestral strain and the evolved strains (A) Biofilm cells formation of the ancestral and evolved strains after 24 h of growth, measured by MTT assay through the OD_570_ value (mean ±s.d.,n = 12). (B and C) Antimicrobial activities of DAP and VAN against WT ancestral MRSA, represented by the minimum concentration needed for inhibiting 90% biofilm formation (minimum biofilm inhibitory concentration, MBIC) (B) and minimum concentration needed for eradicating 50% mature biofilms (minimum biofilm eradication concentration, MBEC) (C) (n = 4). The MBIC_90_ for both DAP and VAN toward ancestral MRSA is 1.25–2.5 μg/mL, while the MBEC_50_ for both DAP and VAN toward ancestral MRSA is 10–20 μg/mL, corresponding to the highlighted area. d, e, Biofilm inhibiting activity of DAP (2.5 μg/mL) (D) and biofilm eradication activity of DAP (20 μg/mL) (E) on the ancestral and evolved strains (mean ±s.d., n = 4). (F and G) Biofilm inhibiting activity of VAN (2.5 μg/mL) (F) and biofilm eradication activity of VAN (20 μg/mL) (G) on the ancestral and evolved strains (mean ±s.d., n = 4). Significance of difference with the ancestral: ns, not significant, ∗p < 0.05, ∗∗p < 0.01, ∗∗∗p < 0.001 (two-tailed t-test with unequal variances).

To reveal the adaptation mechanisms of the MRSA strains toward DAP, we then performed shotgun proteomics to the ancestral and evolved strains, and used two different strategies to analyze our proteomics data (Figure 3A). Combining all replicates, 1764, 1759, 1731, and 1683 distinct proteins were identified for DAP-treated ancestral, S1D14, S2D14, and S3D14 population, respectively, whereas 1702, 1653, 1649, and 1558 distinct proteins were identified for untreated ancestral, S1D14, S2D14, and S3D14 respectively (Figure 3B). These numbers covered around 65% of the total ~2600 proteins in the proteome of common *S*. *aureus* strains, testifying to the depth of our proteome profiling. In addition, the number of surface-associated proteins in the *S*. *aureus* strains identified in this study (>350 cell wall proteins and cytoplasmic membrane proteins in each sample) was much higher than those identified in previous studies (FigureS4) (Ma et al., 2017; Liu et al., 2014; Hempel et al., 2011; Smith et al., 2016). Using the protein expression data, we performed a principal component analysis (PCA) to determine if there were features that distinguished the four strains both under the absence and presence of an antibiotic. We observed that all strains are positioned similarly along PC2 and PC3, but were separated from each other along PC1 (FiguresS5A and S5B). Although they are separated, the 95% confidence interval (CI) ellipse of S1D14 overlaps with S3D14, which indicated that they may share some features. Comparing each sample group across all three axes simultaneously (PC1, PC2, and PC3) also showed that each strain was positioned uniquely and was separated from each other along the three axes, indicating that they had distinct proteome profiles (FigureS5C).

Figure 3C shows the volcano plots of fold changes (compared to the untreated populations) against pvalues (two-tailed *t*test), along with the number of differentially expressed proteins (DEPs) (both down-regulated, fold change below 1.5, and up-regulated, fold change above 1.5). The complete list of DEPs is available in Table S3. To compare the antibiotic response between different strains, we generated a heatmap of the expression level of all DEPs of the ancestral, S1D14, S2D14, and S3D14 populations (Figure 3D). We observed that the protein expression pattern of the resistant strain (S2D14) was more similar to that of the ancestral, while the protein profile of the tolerant strain (S1D14) was more similar to that of the strain with suppressed tolerance (S3D14). Also, as shown by the higher number of DEPs in S3D14 compared to S1D14 (Figure 3C), the additional mutation on S3D14 that reduced their tolerance level seems to further alter the protein expression of the cells. To gain more information on these DEPs, we subjected them to protein-protein interaction network visualization using STRING (Szklarczyk et al., 2016) (Figure 3E). Under sub-MIC DAP treatment, several pathways were up-regulated in both the ancestral and evolved strains, such as the purine metabolism, betaine biosynthesis, and proteins for cell wall formation. These can be viewed as the common responses of MRSA toward DAP stress, which was also observed in *S*. *aureus* by other groups (Müller et al., 2018; Gaupp et al., 2015).

The perturbed network for the resistant strain (S2D14) was similar to that of the ancestral, with the addition of a few enriched pathways, which should be related to the resistance mechanism. One is DNA repair; most of the proteins (RecG, RecJ, RuvB, and UvrC) under this category were up-regulated, except for RecX, which was down-regulated. Although RecA, which has been implicated in *S*. *aureus* SOS-mediated response to various stresses such as exposure to fluoroquinolones (Cirz et al., 2007) and β-lactams (Maiques et al., 2006) is not found to be differentially expressed, we observed that protein RecX, which represses the activity of RecA (Drees et al., 2004; Baharoglu and Mazel, 2014), was down-regulated by 1.5 folds. Another notable up-regulated pathway was the two-component regulatory system, which has been repeatedly linked to DAP resistance and tolerance (Tran et al., 2015; Kuroda et al., 2003; Mwangi et al., 2007; Peyrusson et al., 2020). This system allows bacteria to sense external stimuli and make appropriate protective responses to cell wall defects and cell wall active antibiotics. Here, both sensor histidine kinase KdpD and the putative two-component response regulator PhoP were up-regulated by 1.6 folds. In addition, the resistant strain also has an up-regulated expression of enzyme IIA component of the phosphotransferase system (PTS), which have been reported to be a part of cell-wall active antibiotic stress response (Singh et al., 2001). Taken together, the antibiotic response of the resistant strain (S2D14) is similar overall to the ancestral one, but with the addition of other “beneficial” pathways known to counter the deleterious effect of DAP. This is not surprising given that S2D14 has acquired a well-known resistance mutation in *mprF*, and therefore their observed proteome changes are also likely to be in line with the current knowledge of DAP resistance.

Unlike the ancestral and the resistant strain, the perturbed protein network of S1D14 and S3D14 were much more complicated. In fact, S1D14 and S3D14 shared many differentially expressed pathways, such as ribosomal proteins, DNA repair, cell wall organization, amino acid biosynthesis, pyridoxal phosphate binding, and isoprenoid biosynthesis. They could be important processes for DAP tolerance. It is worth noting that S3D14 with reduced DAP tolerance had more DEPs involved in cell wall organization compared to S1D14, and the expression of their cell-surface associated receptors such as IsdA, IsdB, FnbA, and ClfB, were all down-regulated. Besides, the differential expression of Mur enzymes (MurA, MurB, MurG) that are responsible for the initial step of peptidoglycan biosynthesis was only observed in the ancestral and S3D14 strains, both susceptible toward DAP. Another enzyme, D-alanyl-D-alanine ligase (Ddl), that functions to link two D-alanine to D-alanyl-D-alanine that would later on be integrated to the peptidoglycan by MurF (Jarick et al., 2018), was only up-regulated in S1D14 and S2D14 strain, which is tolerant and resistant to DAP, respectively.

### Differential processes and pathways of the tolerant and resistant strains compared to the ancestral

We compared the proteome profile of the evolved strains (S1D14, S2D14, and S3D14) to that of the ancestral strain as our control to observe alterations in terms of protein expression due to the mutations. Figures 4A and 4B show the volcano plots of fold changes against pvalues (two-tailed *t*test), highlighting the proteins on the evolved strains with different expression levels compared to the ancestral. The list of DEPs is available in Table S4 (in the absence of DAP) and Table S5 (in the presence of DAP). From the number of DEPs, we saw that the resistant strain (S2D14) had the least differences compared to the ancestral, while S3D14 was the most different. We further performed gene ontology (GO) analysis and Kyoto Encyclopedia of Genes and Genomes (KEGG) pathway enrichment study on the DEPs under antibiotic treatment using DAVID (Sherman and Lempicki, 2009) (Figures 4C–4E).

In general, S1D14 and S3D14 had similar differential processes and pathways (compared to the ancestral), such as increased lysine biosynthesis, threonine biosynthesis, glycine, serine, and threonine metabolism, and also tryptophan synthase activity. They both have reduced pathogenesis, purine metabolism, and glutamine metabolic processes. In S1D14 specifically, there was a higher expression in cysteine and methionine metabolism, polysaccharide biosynthetic process, and diaminopimelate biosynthetic process. Besides, they lowered the expression of proteins related to bacterial invasion and infection, cytolysis in other organisms, and they also had a reduced phosphorelay sensor kinase activity through a reduced expression of sensor protein kinase WalK, sensor protein kinase HptS, and sensor histidine kinase SaeS by 1.8, 2.2, and 1.7 folds respectively. In S3D14, we observed a reduced lipid metabolic process, isoleucine metabolic process, and a reduced expression of proteins involved in the two-component system, which is important for *S*. *aureus* tolerance and resistance toward cationic antimicrobial peptides.

**Figure 6:**
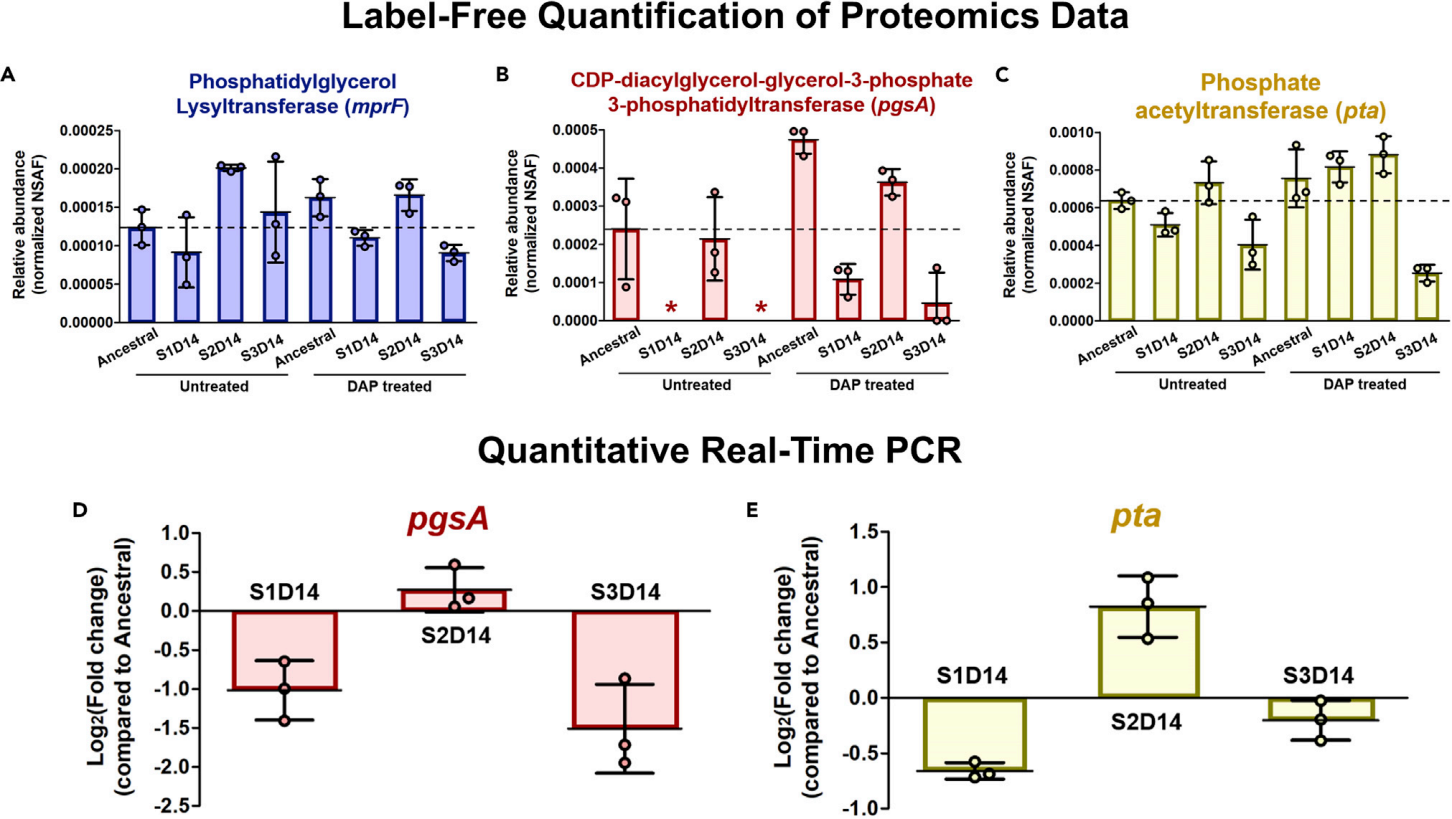
The expression level of the genes that are mutated in the evolved strains (A–C) Relative abundance of the proteins phosphatidylglycerol lysyltransferase (MprF) (A), CDP-diacylglycerol-glycerol-3-phosphate 3-phosphatidyltransferase (PgsA) (B) and phosphate acetyltransferase (Pta) (C) across the ancestral and evolved strains, measured by label-free quantitative proteomics using spectral counting, where the *y*axis is the normalized spectral abundance factor (NSAF) values (mean ± s.d., n = 3). The horizontal dashed line shows the mean expression level of the ancestral strain (without DAP treatment). Asterisks indicate zero NSAF value (not detected by the mass spectrometer). d, e, Fold changes of the gene expression of *pgsA* (D) and *pta* (E) across the evolved strains as compared to the ancestral, measured by RT-qPCR (mean ± s.d., n = 3).

For the resistant strain (S2D14), there were only a few altered processes. Proteins involved in the pathway for cationic antimicrobial peptide (CAMP) resistance were up-regulated compared to the ancestral (Figure 4D), consistent with the observed DAP-resistance phenotype of the strain. These proteins are DltB, ABC transporter ATP-binding protein, and D-alanine-D-alanyl carrier protein ligase, which collectively influence the cell wall properties and transport through the membrane. Interestingly, the expression of mannose-6-phosphate isomerase was much higher in S2D14 than in the ancestral. This has also been observed in another independent study performing comparative transcriptomics between DAP-resistant *S*. *aureus* strain to a susceptible one, where the gene *manA*, expressing mannose-6-phosphate isomerase, is up-regulated by 5-folds in the resistant strain compared with the susceptible strain (Fischer et al., 2011). Besides, the carotenoid biosynthetic process, which is related to the production of the characteristic yellowish-orange pigment of *S*. *aureus* (Liu et al., 2005), was also lower in the resistant strain than the ancestral. Indeed, S2D14 had less orange pigment color than the other strains (FigureS6A). As an independent validation of our proteomics data, we extracted and measured the pigment from the cells by UV/Vis spectrophotometry, and we showed that S2D14 had a shifted absorbance spectrum compared to the ancestral and other strains (FigureS6B). In particular, the OD_450_, which indicates the absorbance of the pigment, was much lower in S2D14 than in the other strains.

Figures 4F and 4G show the Venn diagram of the DEPs from the three evolved strains in the absence and presence of DAP. There were 5 and 8 overlapped DEPs among the three evolved strains in the absence and presence of DAP respectively (Figure 4H). In most overlapping proteins, the direction of differential expression was consistent among the three evolved strains. Interestingly, there were 3 proteins with different expression patterns across the strains. The first one was a lactamase B domain-containing protein (Q2FY29), which was up-regulated in the resistant S2D14 strain but down-regulated in both S1D14 and S3D14 under DAP treatment. Although this protein has not been previously implicated in *S*. *aureus* resistance, its sequence was homologous to β-lactamases, which are enzymes that confer resistance to beta-lactams that target bacterial cell-wall, similar to DAP. Conceivably, the specific up-regulation of this protein in the DAP-resistant strain in the presence of DAP is related to its resistance mechanism. The second one was immunoglobulin-binding protein Sbi (Q2FVK5), which was up-regulated on S2D14 but highly down-regulated in both S1D14 and S3D14 in the absence of DAP. It has been reported that protein Sbi is anchored to the cell envelope by binding to lipoteichoic acid (LTA), and an LTA-defective mutant of *S*. *aureus* had reduced levels of Sbi (Smith et al., 2012). Since the evolved strains had significant alterations in the expression of proteins responsible for cell wall organization (Figure 3E), it may imply that S1D14 and S3D14 have a lower number of LTA molecules anchored in the cell wall than the ancestral strain, while the resistant strain S2D14 has a higher level of LTA, where the protein Sbi can bind to. The last overlapping protein with differential expression in opposite direction among the evolved strains was anthranilate synthase component I (Q2FYR9), whose expression was up-regulated on both S1D14 and S3D14, but down-regulated in S2D14. From the GO analysis (Figures 4C and 4E), tryptophan synthase activity was the most up-regulated molecular function in both S1D14 and S3D14 (fold enrichment above 30 folds), suggesting that the level of tryptophan is possibly higher in S1D14 and S3D14 than in the ancestral strain. Since tryptophan was known to inhibit the activity of anthranilate synthase (Proctor and Kloos, 1973), the increased expression of this protein in S1D14 and S3D14 may be a response to counteract the inhibition of its activity by increased levels of tryptophan.

### Antibiotics are unable to disrupt resistant strain biofilms but can eradicate biofilms of the ancestral and other evolved strains

Since we spotted proteins responsible for *S*. *aureus* infection and biofilm formation among the differentially regulated proteins in the evolved strains, we wanted to know whether the evolved strains have altered biofilm formation and susceptibility toward antibiotics, compared to the ancestral strain. We found that, in general, the evolved strains had lower amounts of biofilm cells after 24 h of growth than the ancestral one, especially for S1D14 and S3D14 (Figure 5A). This was perhaps due to the slower growth of S1D14 and S3D14 than the ancestral strain (Figure 2A). To see the degree of biofilm inhibition and disruption upon antibiotic treatment, we used DAP and VAN to treat the biofilms. The measured minimum biofilm inhibitory concentration (MBIC) of both DAP and VAN toward the ancestral strain was 1.25–2.5 μg/mL, and the minimum biofilm eradication concentration (MBEC) for both DAP and VAN toward the ancestral strain was 10–20 μg/mL (Figures 5B and 5C). Therefore, we used 2.5 μg/mL of DAP and VAN to evaluate the degree of biofilm inhibition and 20 μg/mL of DAP and VAN to evaluate the degree of biofilm eradication on our strains. When the cells were grown with DAP, we could see that the biofilm formation on all strains is completely inhibited at a DAP concentration of 2.5 μg/mL (Figure 5D). Treatment of mature biofilms (grown for 24 h without antibiotic) with DAP showed that the resistant strain (S2D14) biofilm was more resistant toward DAP than the ancestral, while the tolerant strain (S1D14) biofilm was more susceptible toward DAP than the ancestral (Figure 5E). That is, under 20 μg/mL of DAP treatment the biofilm eradication (%) on the resistant strain was only 8%, whereas it was 60% and 86% on the ancestral and tolerant strain, respectively. Interestingly, when the cells were grown in the presence of 2.5 μg/mL VAN, the biofilm formation of the resistant strain was not inhibited while the biofilm formation in the other strains was completely inhibited under the same concentration (Figure 5F). Similarly, under 20 μg/mL of VAN treatment, the extent of biofilm eradication on the resistant strain was much lower than the ancestral, while the tolerant strain biofilm was more susceptible toward VAN than the ancestral (the biofilm eradication was 21%, 82%, and 57% on the resistant, tolerant, and ancestral strains respectively) (Figure 5G). These results were consistent with our GO analysis and pathway enrichment study (Figures 4C–4E), where the tolerant strain possessed lower expression of proteins for *S*. *aureus* infection such as fibronectin-binding protein, d-alanyl carrier protein, immunoglobulin-binding protein Sbi, immunoglobulin G-binding protein A, surface protein G, and leukocidin-like protein, which were implicated in biofilm development of *S*. *aureus* (Lister and Horswill, 2014), while the resistant strain has higher expression of proteins for *S*. *aureus* infection. Therefore, the resistant strain selected through repetitive antibiotic treatment in solution was also more difficult to eradicate when in biofilms, which is a more realistic environment for *S*. *aureus* in clinical patients (Piechota et al., 2018). Interestingly, the biofilms of the tolerant strains did not appear to be more recalcitrant, and in fact were easier to eradicate than the ancestral, susceptible strain.

### The expression of *mprF* is higher on the resistant strain, while *pgsA* and *pta* are lower on the tolerant strains

Using the normalized spectral abundance factor (NSAF) values from our proteomics data, we could estimate the relative expression level of the proteins encoded by the mutated genes in the evolved populations (Figures 6A–6C). The level of the protein phosphatidylglycerollysyltransferase, which was the protein expressed from the *mprF* gene that is mutated on S2D14, was 1.6 folds higher in the S2D14 strain than in the ancestral under normal growth condition (Figure 6A). This agreed with previous reports where *mprF* mutation governing DAP resistance is associated with MprF gain-of-function (Yang et al., 2009; Ernst et al., 2018). CDP-diacylglycerol-glycerol-3-phosphate-3-phosphatidyltransferase, which is the protein expressed from the *pgsA* gene, was not detected in both S1D14 and S3D14 under normal growth condition. Under DAP treatment, the abundance of this protein increased in all strains, but the expression levels in S1D14 and S3D14 strains were still much lower than those in the ancestral and S2D14 (Figure 6B). In the presence of DAP, the expression level of PgsA is 4.3 folds lower in the S1D14 compared with the ancestral strain (Table S5). This indicated that the mutation upstream of the *pgsA* gene that occurs in S1D14 and S3D14 suppresses, but does not abolish, the expression of the protein. The protein phosphate acetyltransferase, encoded by the mutated *pta* gene in S3D14, was found to be expressed by 1.6 folds and 3 folds lower in S3D14 (compared to ancestral) under normal growth condition and under DAP treatment, respectively (Figure 6C). Interestingly, the expression of this protein was also lower in S1D14, but higher in S2D14 under normal growth conditions. Although this protein has no known association with tolerance, differential expression of this protein suggests that the mutation has a material effect on the protein function, which may be connected to the suppressed tolerance phenotype in S3D14.

Although the mutation on *mprF* is already well known to cause an increased expression which led to DAP resistance (Yang et al., 2009), there were no reports about the effect of mutations upstream *pgsA* gene and on the *pta* gene to the expression level of the genes. Therefore, to verify whether the regulation occurs at the transcription or translation level, we performed quantitative real-time PCR (RT-qPCR) on *pgsA* and *pta* genes in the ancestral and evolved strains (Figures 6D and 6E). The results showed that the expression level of *pgsA* was lower in S1D14 and S3D14 than in the ancestral, and the expression level of *pta* was lower in S1D14 and S3D14, but higher in S2D14 than in the ancestral. The expression level of the genes from RT-qPCR followed the same trend as the corresponding proteins, and therefore the regulation of the mutated genes seems to occur upstream of translation.

### The evolved strains possess cell membrane and other cell wall-related modifications

**Figure 7:**
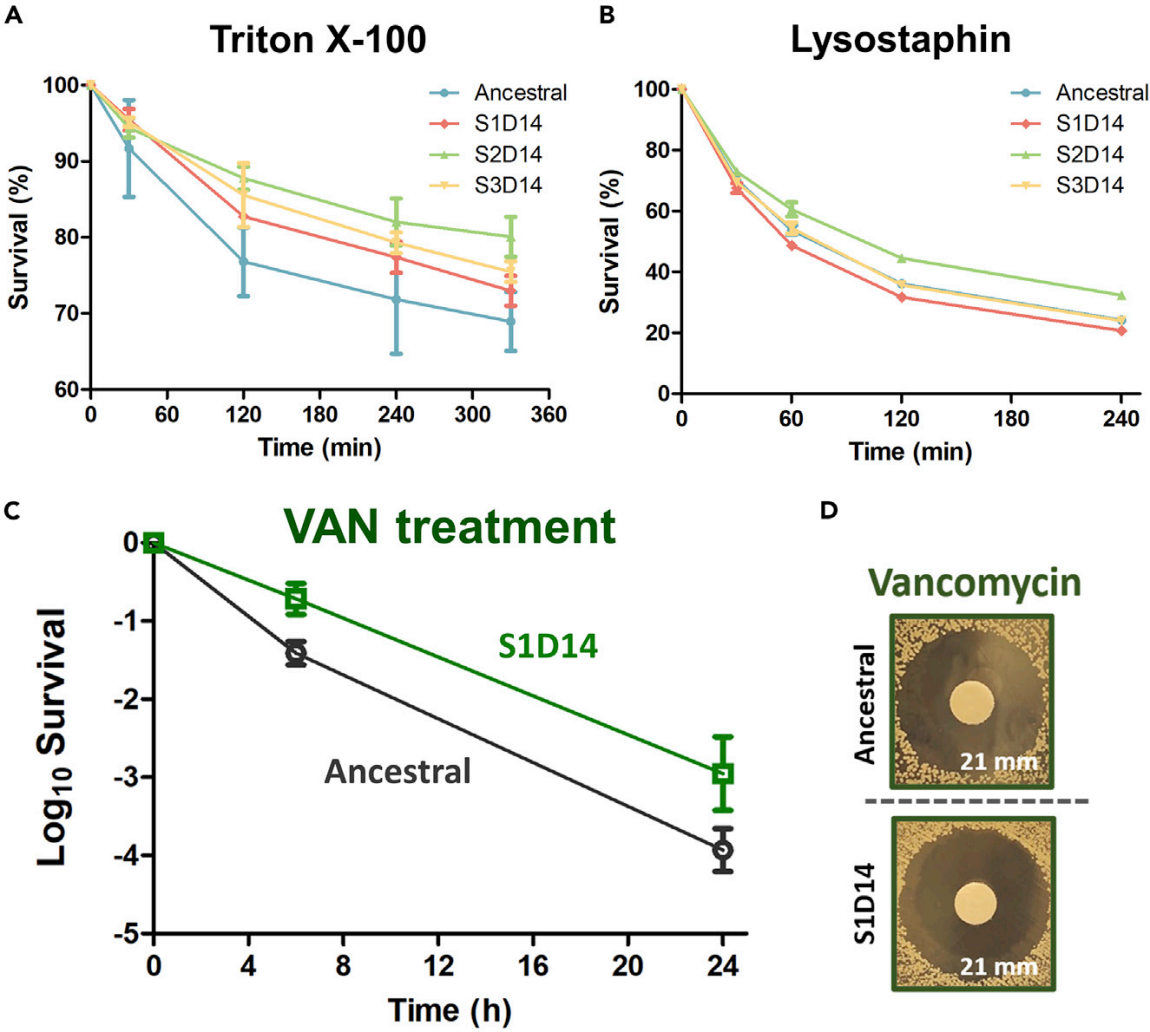
Evolved strains possessed cell membrane and other cell wall-related modifications (A and B) Induced autolysis assay in the ancestral, S1D14, S2D14, and S3D14 strains. Cells were treated with 0.05% Triton X-100 (a) or 1000 ng/mL lysostaphin (B) and incubated at 37°C. Autolysis was measured by monitoring the decrease in OD_600_ over time (mean ± s.d., n = 3). (C) Killing assay of the WT ancestral population and DAP-tolerant S1D14 evolved strain with VAN (30 μg/mL) (mean ± s.d., n = 3). (D) MIC test toward VAN carried out using disc diffusion antibiotic sensitivity testing. The text on the lower right corner marks the diameter of the zone of inhibitions.

Since the mutated genes in the evolved strains code for enzymes for the production of major components of the cell envelope, and indeed the protein levels were found to be altered, we want to investigate whether the evolved strains possess modifications in their cell membrane and peptidoglycans. We performed induced-autolysis assay in the presence of Triton X-100 (a detergent that induces cellular lysis by disrupting the plasma membrane through the disruption of the hydrogen bonding in the lipid bilayer) and lysostaphin (an endopeptidase that cleaves the cross-linking pentaglycine bridges on the peptidoglycan layer) (Figures 7A and 7B). First, we observed that all evolved populations had higher survival toward Triton X-100 than the ancestral, suggesting that DAP tolerance and resistance in the evolved populations may result from alterations of the cell membrane, perhaps by modulating the abundance of membrane-associated proteins and thus affecting membrane stability and fluidity. Under lysostaphin treatment, however, only the resistant strain has a higher survival compared to the ancestral, indicating that only the resistant strain possesses modifications in the cell wall peptidoglycans.

Besides DAP, another antibiotic that is commonly used in clinics to treat MRSA is VAN, which inhibits bacterial cell wall synthesis. When our DAP-tolerant evolved strain (S1D14) was treated with VAN, they showed cross-tolerance toward VAN (Figure 7C), without a difference in MIC toward VAN compared to the ancestral (Figure 7D). This indicates that the tolerant strain possesses other cell wall changes which may also reduce the effectiveness of VAN, but the peptidoglycan does not seem to be involved (Figure 7B). In addition, our proteomics data also shows that many proteins with altered expression in the evolved strains (compared to the ancestral) were in fact cell membrane and cell wall proteins, which cover ~25% of all DEPs (Tables S3 and S4).

## Discussion

Through this study, we demonstrated how different antibiotic treatment schemes might affect the evolution of tolerance and resistance in bacterial populations. To our knowledge, this is the first attempt to conduct ALE experiments with antibiotic combination treatments in MRSA, especially using antibiotic treatment schemes mimicking clinical conditions. This study extended previous work investigating the effect of drug combinations toward the development of resistance in *E*. *coli* (Yeh et al., 2006; Chait et al., 2007; Hegreness et al., 2008), ALE experiments performed in *S*. *aureus* but not using drug combinations (Mechler et al., 2015), and MRSA strains evolved in the host under combinatorial drug treatment in the clinic (Liu et al., 2020). In this study, we discovered that differences in the treatment conditions can lead to different tolerance/resistance phenotypes. Two weeks of intermittent DAP treatment led to DAP-tolerant strain (S1D14), while DAP/RIF treatment led to a DAP-resistant strain (S2D14). Interestingly, adding RIF to the treatment regime after one week of DAP treatment led to reduced tolerance (S3D14). Overall, the apparent ease of evolution of distinct strains with different phenotypes in such a short time highlights the diverse evolutionary pathways available to the bacteria to develop tolerance and resistance, and the benefits and costs associated with the specific treatment conditions may bias the selection of one mutant strain over another, showing the complex competition dynamics among emerging mutants (Windels et al., 2020).

Besides, the mutations detected in our evolved strains have been previously reported in clinical isolates and laboratory strains. For instance, mutations at various sites in *mprF* have been widely recognized to govern DAP resistance in *S*. *aureus* strains (Ernst et al., 2018; Friedman et al., 2006; Sabat et al., 2018). Gain-of-function nonsynonymous mutations in the *mprF* gene (codes for phosphatidylglycerollysyltransferase) have been known to cause increased production of positively charged lysyl-phosphatidylglycerol (LysPG), which is a major bacterial membrane component. LysPG, when translocated to the outer cell membrane, would enhance the net positive surface charge and reduce DAP binding (Yang et al., 2009). Indeed, we observed a higher expression of phosphatidylglycerollysyltransferase in the resistant strain S2D14 than in the ancestral (Figure 6A), which is the protein expressed from the *mprF* gene. Another mutation that is known to cause DAP resistance is in the *pgsA* gene, which was reported to occur not only in *S*. *aureus* (Peleg et al., 2012; Hines et al., 2017) but also in other organisms such as *Bacillus subtilis* (Hachmann et al., 2011) and *Streptococcus mitis* and *Streptococcus oralis* (Tran et al., 2019). This resistance phenotype has been ascribed to the impairment of PgsA function that causes the reduction in the amount of negatively charged phosphatidylglycerol (PG) in the membrane, affecting the fluidity of the membrane and subsequently reduce DAP binding. Peleg et al. reported six mutations on *S*. *aureus pgsA* gene that led to a 4-fold increase in the MIC toward DAP (Peleg et al., 2012). The mutation in our tolerant strain, however, did not occur in the coding region of the *pgsA* gene, but 9 base pairs before the start codon. This mutation caused a reduced expression of *pgsA* and the corresponding protein, CDP-diacylglycerol-glycerol-3-phosphate-3-phosphatidyltransferase (Figures 6B and 6D), and apparently led to a DAP tolerance phenotype, which was indicated by a higher value of MDK_99_, but no increase in the MIC. Altogether, our result seems to suggest that mutations on the same gene (*pgsA*) can lead to different survival strategies toward DAP (tolerance or resistance), depending on the actual site. It has been proposed that tolerance mutations occur more frequently than resistance mutations due to the larger target size (Levin-Reisman et al., 2017). Perhaps, resistance mutations need to be at a certain site of the gene (as shown by Peleg et al. where almost all of the mutations on *pgsA* are located in the transmembrane domains (Peleg et al., 2012)), but many other mutations in or near the gene that affects the expression of the gene/protein can lead to tolerance.

Finally, the suppressed tolerance observed on S3D14 may be linked to an additional mutation in the pta gene, expressing phosphate acetyltransferase for the synthesis of acetyl phosphate from acetyl-CoA. Since RIF was added to the treatment regime in the second week (scheme 3), the additional mutation on S3D14 may offer competitive advantages toward RIF (as hinted from the slight increase in the MIC toward RIF in the second week, Figure 1E**; right**), although they somehow compromise the DAP-tolerance conferred by the previous mutation (upstream the *pgsA* gene) that emerged during single DAP treatment. These demonstrated how antibiotic treatment could affect bacterial populations very differently depending on their initial genotype. DAP/RIF combination treatment to the susceptible ancestral strain led to the *mprF* mutation that confers DAP resistance, while the same combination treatment applied to a strain already tolerant to DAP led to additional mutations that increase DAP susceptibility. We observed that the transcript level of the *pta* gene and the abundance of the corresponding protein, phosphate acetyltransferase, was lower in S3D14 than in the ancestral (Figures 6C and 6E). Although there is no literature that explicitly associates this gene with DAP tolerance or resistance, it has been shown that acetyl phosphate is involved in the phosphorylation of LytR (Patel and Golemi-Kotra, 2015), a member of the two-component system that regulates genes controlling cell apoptosis, autolysin activity, biofilm formation, and is also involved in the adaptation of *S*. *aureus* to CAMPs (Patton et al., 2006; Sharma-Kuinkel et al., 2009). LytR is activated through phosphorylation by a membrane-bound histidine kinase LytS, but it was shown that acetyl phosphate is able to phosphorylate LytR at a rate that is 2-folds faster than phosphorylation by LytS, suggesting that phosphorylation via acetyl phosphate is a more efficient signaling pathway with faster kinetics. Thus, depletion of acetyl phosphate in S3D14 could in turn affect the regulation of LytR. It is also worth noting that the expression level of phosphate acetyltransferase and the *pta* gene is significantly higher in the resistant strain (Figures 6C and 6E), which reinforces the notion that this protein may be important for the cell’s adaptation toward DAP.

Our proteomics data also confirm what is generally known about the resistance and tolerance mechanisms, where tolerance mutations are more upstream and may be better considered as the result of a perturbed biological network, while resistance mutations are directly related to the action mechanism of the antibiotic. Our resistant strain had a less perturbed proteome profile upon antibiotic challenge as it only needed to activate certain resistance-associated mechanisms (such as up-regulation of proteins involved in the CAMP resistance, activation of the two-component system, DNA repair machinery, lower carotenoid biosynthesis), but the tolerant strain did not have such a direct counter, so it required a more complicated antibiotic response to survive. This was evident from a lower observed growth rate, and a large number of differentially regulated processes measured by proteomics, including reduced ribosomal proteins and phosphorelay sensor kinase activity, increased tryptophan synthase activity, and cell wall modulation. Besides, we also showed that both of the resistant and tolerant strains possess cell membrane and other cell wall-related modifications (Figure 7), which might affect their survival under DAP treatment. These are consistent with previous reports which stated that the mechanisms of DAP resistance on S. *aureus* are diverse and complex, and it involves perturbations mainly in the cell membrane, but also in the cell wall (Peleg et al., 2012; Bayer et al., 2013).

As we have shown in the Results section, our proteomics data is very rich and is waiting to be explored. Right now, many of the DEPs are not previously known to be related to tolerance. Besides, compared to *Escherichia coli*, which has been more thoroughly studied in tolerance research, there are gaping holes in our current understanding of *S*. *aureus* tolerance, despite its clinical importance. As our knowledge of this biological system advances in the future, the data can be re-analyzed for new insights. In our view, proteomics is the perfect tool for studying antibiotic tolerance, which by its nature needs to be investigated from a systems point of view (Sulaiman and Lam, 2019; Sulaiman et al., 2018). Advancing technology will soon enable us to observe the cellular changes at the systems level in real-time and in finer resolution, as the bacteria adapt to different antibiotic treatments. Combined with the ALE strategy to generate diverse tolerant mutants, such large-scale studies should be our most direct path to comprehensively mapping the so-called “tolerome” (Sulaiman and Lam, 2021; Brauner et al., 2016) and clarifying the mechanisms of tolerance. Overall, we believe that this study is a clear step forward in our effort to survey the landscape of genetic and metabolic changes that govern tolerance in *S*. *aureus*.

### Limitations of the study

In this study, we employed ALE to generate MRSA strains with distinct tolerance and resistance phenotypes, which were then cross-compared by proteomics to reveal key proteins and pathways relevant to mechanisms of tolerance and resistance. We did not investigate the evolutionary pathways or the population dynamics during the ALE experiments, so this study does not address the question of when or how tolerance or resistance emerges. In addition, proteomics by nature provides a bird’s eye view into the physiology of the cells but is not well-suited to elucidate the step-by-step molecular mechanisms underlying these phenomena. It helps lead us to useful hypotheses, such as the possible tolerance-suppressing role of *pta* in the S3D14 strain, which can then be tested by future follow-up experiments involving genetic and/or biochemical manipulation. Finally, this study demonstrated an effective strategy for surveying the “tolerome” of MRSA, namely, by mining for tolerant strains by ALE and studying their responses to antibiotic assault by proteomics. However, many of the DEPs detected are not known to be related to tolerance, and some remain uncharacterized or hypothetical in protein databases due to the currently limited knowledge about this organism and tolerance in general. As the tolerome is further mapped and our understanding advances with more experiments, which will require the whole community’s effort, our high-quality proteomics data can be readily reanalyzed for more insights.

## STAR★Methods

### Key resource table

**Table:** 

REAGENT or RESOURCE	SOURCE	IDENTIFIER
**Bacterial and virus strains**		

Methicillin-resistant *Staphylococcus aureus* (MRSA)	ATCC	ATCC 43300
Evolved MRSA strains, see Table S1	This paper	see Table S1

**Chemicals, peptides, and recombinant proteins**		

Mueller-Hinton (MH) broth	Sigma Aldrich	Cat#90922-500G
Mueller-Hinton (MH) agar	Sigma Aldrich	Cat#70191-500G
Lysogeny Broth (LB)	Invitrogen	Cat#12780-052
Lysostaphin	Sigma Aldrich	Cat# L9043-5MG; CAS:9011-93-2
Triton X-100	Sigma Aldrich	Cat#X100-500ML; CAS:9002-93-1
Daptomycin	Sigma Aldrich	Cat#SBR00014-0.5ML; CAS:103060-53-3
Rifampin	Sigma Aldrich	Cat#R3501-1G; CAS:13292-46-1
Vancomycin	Sigma Aldrich	Cat#V2002-1G; CAS:1404-93-9
Bovine Serum Albumin Standard	Thermo Scientific	Cat#23209
Urea	Sigma Aldrich	Cat#U5378-100G; CAS:57-13-6
Sodium Chloride	IBI Scientific	Cat#89140-860; CAS:7647-14-5
Guanidine hydrochloride	Sigma Aldrich	Cat#G3272-100G; CAS:50-01-1
Dithiotreitol	Sigma Aldrich	Cat#43815-5G; CAS: 3483-12-3
Iodoacetamide	Sigma Aldrich	Cat#I6125-5G; CAS:144-48-9
Ammonium bicarbonate	Sigma Aldrich	Cat#A6141-25G; CAS:1066-33-7
Trypsin	Promega	Cat#5111
Formic acid	Sigma Aldrich	Cat#56302-10X1ML-F
Acetonitrile	J.T. Baker	Cat#JT9829-3
Glucose	Sigma Aldrich	Cat#G7021-1KG; CAS: 50-99-7
Dimethyl sulfoxide (DMSO)	Sigma Aldrich	Cat#8418-250ML; CAS:67-68-5
Methanol	J.T. Baker	Cat#JT9830-3
Trizma base	Sigma Aldrich	Cat#1503-100G; CAS:77-86-1
Hydrochloric acid	Sigma Aldrich	Cat#320331-2.5L; CAS:7647-01-0
3-(4,5-dimethylthiazol-2-yl)-2,5-diphenyl tetrazolium bromide (MTT)	Sigma Aldrich	Cat#M2128-1G; CAS:298-3-1

**Critical commercial assays**		

LIVE/DEAD *Bac*Light bacterial viability kit	Invitrogen	Cat#L7007
E.Z.N.A. Stool DNA kit	Omega Bio-tek	Cat#D4015
RNAprotect® Cell Reagent	Qiagen	Cat#76526
RNeasy PowerBiofilm Kit	Qiagen	Cat#25000-50
RevertAid H Minus First Strand cDNA Synthesis Kit	Thermo Scientific	Cat#K1632
SYBR® Green RT-PCR Reagents Kit	Applied Biosystems	Cat#4334973
Pierce™ BCA Protein Assay Kit	Thermo Scientific	Cat#23225
Amicon® filter device	Millipore	Cat#UFC501096
C18 reverse-phase ZipTip	Millipore	Cat#ZTC18S096

**Deposited data**		

Mass spectrometry proteomics data submitted to ProteomeXchange	This study	PXD021667
Whole genome sequence data submitted to BioProject database (NCBI)	This study	PRJNA660918

**Oligonucleotides**		

Primers for RT-qPCR, see Table S6	(McClary et al., 2017), (Chen et al., 2018), and this paper	N/A

**Software and algorithms**		

BWA mapper V0.7.17	(Li and Durbin, 2009)	http://bio-bwa.sourceforge.net/
Integrative Genomics Viewer V2.9.4	(Robinson et al., 2011)	https://software.broadinstitute.org/software/igv/
SAMTOOLS V1.9	(Li et al., 2009)	http://www.htslib.org/download/
Snippy V4.6.0	(Seemann, 2015)	https://github.com/tseemann/snippy
BreakDancer V1.4.5	(Chen et al., 2009)	http://breakdancer.sourceforge.net/
Bruker Compass DataAnalysis version 5.2	Bruker	https://www.bruker.com/service/support-upgrades/software-downloads/mass-spectrometry.html
Msconvert of the ProteoWizard	(Kessner et al., 2008)	http://proteowizard.sourceforge.net/tools.shtml
Comet (version 2016.01 rev.2)	(Eng et al., 2013)	http://comet-ms.sourceforge.net/
GeneMark version 3.25	(Lukashin and Borodovsky, 1998)	http://exon.gatech.edu/GeneMark/
BLASTp (version 2.7.1)	NCBI	https://blast.ncbi.nlm.nih.gov/Blast.cgi?PAGE=Proteins
Trans-Proteomics Pipeline (TPP)	(Deutsch et al., 2010)	http://tools.proteomecenter.org/wiki/index.php?title=Software:TPP
PSORTb version 3.0.2	(Yu et al., 2010)	https://www.psort.org/psortb/
STRING version 11.0	(Szklarczyk et al., 2016)	https://string-db.org/
DAVID version 6.8	(Sherman and Lempicki, 2009)	https://david.ncifcrf.gov/

### Resource availability

#### Lead contact

Further information and requests for resources and reagents should be directed to and will be fulfilled by the Lead Contact, Henry Lam (kehlam@ust.hk).

#### Materials availability

This study did not generate new unique reagents.

### Experimental model and subject details

#### Bacterial strains and growth conditions

Bacterial strain used for the evolution experiment is methicillin-resistant *S*. *aureus* (MRSA) ATCC 43300. Exponential phase cultures were prepared by incubating a 1:200 diluted overnight culture in cation-adjusted Mueller-Hinton (MH) broth until OD_600_ reached ~0.1 at 37⁰C with shaking. MH broth used in this study is supplemented with Ca^2+^ to a final concentration of 50 mg/L to mimic the physiological levels of calcium ions, which is important for the concentration-dependent bactericidal activity of daptomycin (Safdar et al., 2004; Steenbergen et al., 2005; Silverman et al., 2003). MH agar was used for colony counts.

To get rifampin-resistant mutants, ~$10^{9}$ bacteria were plated into MH agar plates supplemented with 1 μg/ml rifampin and incubated at 37⁰C overnight. The MIC of the colonies that appeared were measured to confirm the resistant phenotype, and the genomic DNA of the colonies was subjected to whole-genome sequencing to identify the mutations. All rifampin-resistant mutants isolated from the WT ancestral, S1D7, and S2D7 background harbor mutations in the *rpoB* gene with the same single point mutation (Table S1).

### Method details

#### Evolution experiment

Exponential phase culture was prepared by incubating a 1:200 diluted overnight culture in 1 mL cation-adjusted Mueller-Hinton (MH) broth for ~3 h at 37⁰C with shaking at 250 rpm. For the treatment, exponential phase culture was exposed to either 10 μg/ml daptomycin (DAP), or 10 μg/ml daptomycin and 1 μg/ml rifampin (RIF) combination for 2 h. The antibiotic-containing medium was removed by washing three times in MH broth (10 min centrifugation at 4,500 *g*), and the cells were resuspended in 1 ml fresh MH and grown overnight at 37⁰C with shaking. The concentration of daptomycin and rifampin was chosen to be similar to those from previous studies (Barros et al., 2019; Liu et al., 2020), and the duration of treatment was chosen based on the biphasic killing curve to ensure that the remaining populations after treatment are all persisters.

There are three treatment schemes performed in this study (Figure 1A). In the first scheme, MRSA population was treated with DAP for 2 weeks. In the second scheme, MRSA population was treated with DAP/RIF combination for 2 weeks and in the third scheme, MRSA population was treated with DAP for one week, followed by DAP/RIF combination for one week. Note that due to the random nature of mutagenesis in such laboratory evolution experiments and to ensure comparable results between the first and third schemes, the population subjected to the initial week of DAP treatment for schemes 1 and 3 are the same population. After the first week, the population was divided into two, one subjected to another week of DAP treatment (scheme 1), and the other subjected to another week of DAP/RIF combination treatment (scheme 3).

#### Tolerance and resistance assay

The concentration of antibiotics used for treatment is 10 μg/ml and 1 μg/ml for daptomycin and rifampin, respectively. For cross-tolerance assay towards vancomycin, the concentration used for treatment is 30 μg/ml. To assess cell viability after antibiotic treatment, the number of survivors were counted by serially diluting cultures in MH broth, plating 100 μl on MH agar and spread plates. The minimum duration of killing 99% of the population (MDK_99_) values were extracted from the time-kill curve generated by measuring the survival % of the population after 1 h and 3 h of treatment.

The MICs of the population were recorded by broth macrodilution method and disc diffusion for visualization. For broth microdilution, the MIC was determined by incubating ~$5\cdot 10^{5}$ exponential phase bacteria in MH medium overnight with various concentrations of antibiotics. The MIC value was determined as the lowest concentration without growth, according to EUCAST guidelines. The disc diffusion test was performed according to the standard EUCAST susceptibility test guideline where inoculum suspension equivalent to a 0.5 McFarland standard ($1-2\cdot 10^{8}$CFU/ml) was spread on MH agar applied with antimicrobial disk (containing 10 μg daptomycin, 5 μg rifampin, or 30 μg vancomycin), and incubated at 35⁰C for 20 h (Jorgensen and Turnidge, 2015).

#### Epifluorescence microscopy

Epifluorescence microscopy was used to observe the ancestral and evolved strains during daptomycin treatment. Before and after 1 h of treatment, 100 μl of the cells are taken, centrifuged, and resuspended in 500 μl of 0.85% NaCl. Cultures were stained with LIVE/DEAD *Bac*Light bacterial viability kit (Molecular Probes) according to the manufacturer’s standard protocol. 1.5 μl of dye mixture containing SYTO 9 (1.67 mM) and propidium iodide (10 mM) was added to the 500 μl culture and incubated in the dark for 10 min. Stained cells were viewed with fluorescence microscope (Eclipse Ni-U Upright Microscope) with appropriate filter sets. Images were captured with Nikon DS-Fi3 and associated software (NIS-Elements Ver. 5.00).

#### Genomic extraction and whole-genome sequencing

WT strain and five isolates from the evolution experiment (S1D7, S1D14, S2D7, S2D14, and S3D14) were subjected to whole-genome sequencing. These evolved strains were selected to be sequenced since the killing curves, MDK_99_, and MIC of the population are not changing any more upon further treatment, suggesting that the sequenced isolates are already established and comprise the majority of the population. Strains were grown from a single colony to OD_600_ of 0.3, and the cell pellets were sent to BGI for genomic DNA extraction and sequencing. Genomic DNA was extracted using E.Z.N.A. Stool DNA kit (Omega Bio-tek) with BGI modifications, and subsequently detected and quantified by the agarose gel electrophoresis and a Qubit fluorometer. The genomic DNA was subjected for paired-ends Illumina sequencing at 2 × 150 bp read length and 350 bp insert size. Sequencing quality was affirmed using FastQC algorithm. The sequenced data were filtered, adapter sequence and low-quality data were removed, resulting in the clean data used for subsequent analysis. Specific processing steps are as follows: Remove reads whose low-quality nucleotides (Q-value ≤38) exceed a certain threshold (40 bp by default), eliminate reads which contain N nucleotides exceeding a certain threshold (10 bp by default), eliminate reads whose overlap with the adapter exceeds a certain threshold (15 bp by default), and finally filter the duplication. The clean bases of each sample are ~1.4 billion bp for WT ancestral, S1D7, S1D14, S2D7, and ~1.2 billion bp for S2D14 and S3D14, and the clean reads are ~9.5 million reads for WT ancestral, S1D7, S1D14, S2D7, and ~8 million reads for S2D14 and S3D14. The WGS raw data were submitted and are accessible in BioProject PRJNA660918.

#### Competition assay

Around 10^3^ rifampin-resistant derivatives (*rpoB* H481Y) were mixed with around 10^6^ of their sensitive strains, and killing assays were performed on the mixed culture under combination treatment (10 μg/ml daptomycin and 1 μg/ml rifampin) for 1 h. The cells were washed twice to remove antibiotics, and the culture was regrown in fresh MH broth overnight at 37 °C with shaking. The survival of rifampin-resistant mutants was evaluated by plating on MH agar containing rifampin antibiotic (1 μg/ml).

#### Pigment quantification

To extract the staphyloxanthin pigment and other intermediate carotenoids, ancestral and evolved strains (S1D14, S2D14, and S3D14) were grown at 37°C for 24 h. Cells were centrifuged and washed twice with phosphate-buffered saline (PBS). An equal amount of cells were resuspended in 750 μl methanol, heated at 55°C for 30 min with shaking. The methanol extract was subsequently cooled and centrifuged, and the supernatant was taken. Pigment content was quantified spectrophotometrically by measuring the absorbance spectrum of the methanol extract and also by measuring the OD_450_ (Mishra et al., 2011).

#### Induced autolysis assay

The induced autolysis assay with Triton X-100 and lysostaphin was performed following previously established protocol (Barros et al., 2019; Gründling et al., 2006). For Triton X-100-induced autolysis, cells were grown to an OD_600_ ~ 0.7, chilled on ice, and harvested by centrifugation. Cells were washed with ice-cold water and then resuspended in 50 mM Tris-HCl (pH 7.5) containing 0.05% Triton X-100 (Sigma Aldrich). For lysostaphin-induced autolysis, cells were also grown to an OD_600_ ~ 0.7 and harvested by centrifugation. Cells were washed with water and resuspended in PBS supplemented with 1000 ng/ml lysostaphin (Sigma Aldrich). Cells were then incubated at 37°C and autolysis was measured by monitoring the decrease in OD_600_ over time.

#### Biofilm assay

Biofilm assay was performed following previously established protocol (Yin et al., 2019; Nair et al., 2016). To see the biofilm formation of the cells and the degree of biofilm inhibition, overnight culture of WT ancestral and evolved strains was diluted into approximately 10^7^ CFU/mL with Lysogeny Broth (LB) and 0.5% glucose (supplemented with Ca^2+^ to a final concentration of 50 mg/L) and treated with various concentrations of antibiotic in 96-well cell culture plates. Plates were then incubated at 37°C for 24 h and rinsed twice with 1×PBS to remove non-adhering and planktonic cells. After rinsing, MTT staining assay was conducted to measure viable cells in the biofilms since MTT can react with activated succinate dehydrogenase in viable cell mitochondria to form blue-violet formazan, which can be measured at 570 nm after dissolving in DMSO. The degree of biofilm inhibition (%) is calculated by comparing the OD_570_ values of the antibiotic-treated cells with the OD_570_ values of the cells grown without antibiotics. To see the degree of biofilm eradication, an overnight culture of ancestral and evolved strains were incubated for 24 h in 96-well cell culture plates to form biofilms (without the addition of antibiotics). The formed biofilm was rinsed twice with 1×PBS and challenged with antibiotic at a series of concentrations and incubated for another 24 h at 37°C. After incubation, each well was rinsed twice with 1×PBS, and the MTT assay was conducted to measure viable cells in the remaining biofilm. The degree of biofilm eradication (%) is calculated by comparing the OD_570_ values of the antibiotic-treated biofilms with the OD_570_ values of the biofilms without antibiotic treatment.

The lowest concentrations of DAP and VAN that resulted in decreases of at least 90% and 50% in OD_570_ of the WT ancestral cells were recorded as minimum biofilm inhibitory concentration (MBIC) and minimum biofilm eradication concentration (MBEC), respectively.

#### RT-qPCR

The expression of *pgsA* and *pta* was quantified by real-time PCR. Cells were cultured in the same condition as the proteomics experiments. The primers for real-time PCR were listed in Table S6. PCR product length was set within the range of 100-200 bp, and the optimal melting temperature was set to be 60°C. The gene *gyrB* was selected as the internal standard gene, as its expression level remains almost constant across all samples.

In order to stabilize RNA, RNAprotect® (Qiagen, California, U.S.A) was added to the cells immediately after the cells being collected. Total RNA was extracted using RNeasy PowerBiofilm Kit (Qiagen, California, U.S.A) according to the manufacturer’s instruction. RT-qPCR was performed in two steps. First, RNA was reverse transcribed to cDNA using RevertAid H Minus First Strand cDNA Synthesis Kit after the removal of genomic DNA using DNase I (Thermo Fisher Scientific Inc., Waltham, Massachusetts, U.S.A). Then, RT-PCR was conducted on LightCycler 480 II (Roche) using SYBR® Green RT-PCR Reagents Kit (Applied Biosystems) with the following procedures: (1) polymerase activation at 95 °C for 10 min, and (2) annealing and extending at 60°C for 1 min with a total of 40 cycles. The specificity of primer pairs for the PCR amplification was checked by the melting curve analysis which was performed immediately after amplification.

#### Sample preparation for proteomics

For proteomics analysis, exponential phase ancestral, S1D14, S2D14, and S3D14 strains were treated with sub-MIC doses of daptomycin (0.25 μg/ml) for 1 h, which should enable the populations to elicit an antibiotic response (Liu et al., 2014, 2016). Exponential phase cells before antibiotic treatment were also collected. Two different strategies are used for the proteomics analysis: (1) First, we compared the proteome profile of each strain before and after DAP treatment to obtain strain-specific antibiotic response towards DAP stress. (2) We also compared the proteome profile of the evolved strains to the ancestral strain as control, both in the presence and absence of DAP, to obtain the differences in protein expression between strains under normal conditions or under antibiotic challenge. For all proteomics experiments, three biological replicates were performed for each sample including the control sample.

The cell pellet was suspended in 350 μL of lysis buffer (8M Urea, 50 mM Tris-HCl pH 8.0), froze in liquid nitrogen, and sonicated for 12 min. The sample was centrifuged (16,000 ×*g* for 10 min) to remove cell debris and insoluble materials. An aliquot of the sample was taken for BCA protein assay (Pierce™ BCA Protein Assay Kit). After protein quantification, the sample was reduced by dithiothreitol (DTT) (0.1 M final concentration) at 37 ⁰C for 1 h. For shotgun proteomics, 150 μg of proteins were mixed with up to 250 μl of the exchange buffer (6M Urea, 50 mM Tris-HCl pH 8.0, 600 mM guanidine HCl), transferred to Amicon® filter device (Millipore, Darmstadt, Germany), and centrifuged (14,000 ×*g* for 20 min). The proteins in the filter device were alkylated with iodoacetamide (IAA, 50 mM in exchange buffer) in dark for 20 min, and then centrifuged (14,000 ×*g* for 20 min). To dilute the urea concentration, 250 μl of 50 mM ammonium bicarbonate was added to the filter device and centrifuged (14,000 ×*g* for 20 min). This step was repeated once. Proteins were digested by sequencing-grade modified trypsin (1:50 w/w, Promega, Madison, WI) for 12 h at 37⁰C. Then, the sample was acidified with 10% formic acid to a final concentration of 0.1% (v/v) and centrifuged for 16,000 ×*g* for 5 min. Finally, the samples were desalted by C18 reverse-phase ZipTip (Millipore, Darmstadt, Germany) and dried with SpeedVac (Eppendorf, Hamburg, Germany) for 30 min.

#### Liquid chromatography

The samples were reconstituted in 25 μl water:acetonitrile:formic acid in a 97.9:2:0.1 ratio (v/v/v), and processed through Bruker nanoElute Ultra High-Performance Liquid Chromatography (UHPLC) (Bruker Daltonics, Bremen, Germany) coupled to a hybrid trapped ion mobility-quadrupole time-of-flight mass spectrometer (TimsTOF Pro, Bruker Daltonics, Bremen, Germany) via a nano-electrospray ion source (Captive Spray, Bruker Daltonics). A volume of 1 μl (approximately 200 ng of the protein digest) was injected into the UHPLC system and separated on an IonOpticks 25cm Aurora Series Emitter column with Captive Spray Insert (250 mm × 75 μm internal diameter, 120 Å pore size, 1.6 μm particle size C18) at a flow rate of 0.3 μl/min. The mobile phase composition is 0.1% formic acid in water for solvent A, and 0.1% formic acid in acetonitrile for solvent B. The gradient was applied from 2 to 5% of solvent B for 0.5 min, from 5 to 30% of solvent B for 26.5 min, and then from 30 to 95% of solvent B for 0.5 min. In the end, the mobile phase was kept at 95% of solvent B for 0.5 min, and then decreased to 2% of solvent B for 0.1 min. 2 minutes equilibration with 2% of solvent B was applied before the next injection.

#### TimsTOF pro mass spectrometer

A detailed description of the mass spectrometer could be seen in previous studies (Meier et al., 2015, 2018). Briefly, ions from the Captive Spray Ion source enter the first vacuum stage where they are deflected by 90° and accumulated in the front part of a dual Trapped Ion Mobility Spectrometry (TIMS) analyzer. An RF potential of 300 V_pp_ is applied to radially trap the ion cloud. After the initial accumulation step, ions are transferred to the second region of the TIMS analyzer to perform ion mobility analysis in parallel. In both parts of the TIMS analyzer, the RF voltage is superimposed by an increasing longitudinal electrical field gradient, such that ions in the tunnel are dragged by the incoming gas flow from the source and repulsed by the electrical field at the same time. By ramping down the electrical field, it releases ions from the TIMS analyzer in order of their ion mobility for QTOF mass analysis. The dual TIMS setup enables operation at 100% duty cycle when accumulation and ramp times are kept equal. Here, we set the accumulation and ramp time to 100 ms each and recorded mass spectra in the range from m/z 100–1700 using the positive electrospray mode. The ion mobility was scanned from 0.85 to 1.30 Vs/cm^2^. The quadrupole isolation width was set to 2 Th for m/z < 700 and 3 Th for m/z > 700, and the collision energy was linearly increased from 27 eV to 45 eV as a function of increasing ion mobility. The overall acquisition cycle of 0.53 s comprised one full TIMS-MS scan and four Parallel Accumulation-Serial Fragmentation (PASEF) MS/ MS scans. Low-abundance precursor ions with an intensity above a threshold of 2,500 counts but below a target value of 20,000 counts were repeatedly scheduled and otherwise dynamically excluded for 0.4 min. The TIMS dimension was calibrated linearly using three selected ions from the Agilent Es LC/MS tuning mix [m/z, 1/K0: (622.0289, 0.9848 Vs cm^−2^), (922.0097, 1.1895 Vs cm^−2^), and (1221,9906, 1.3820 Vs cm^−2^)] in positive mode.

### Quantification and statistical analysis

#### Statistical analysis for survival measurement

For viability tests to measure survival % under antibiotic treatments (and autolysis assay) for planktonic cells and biofilm cells, all replicates were independent biological replicates. Significance was assessed using two-tailed *t*-tests with unequal variances on log-transformed values and a p-value threshold of 0.05.

#### Whole genome sequencing data analysis

We performed a genomic comparison between the ancestral and evolved strains to the reference genome. The variation information of the sample and the reference is obtained by aligning the sample reads with the reference genome (MRSA ATCC 43300 genome downloaded from ATCC website, September 2020) using BWA mapper V0.7.17 (Li and Durbin, 2009). The parameters of BWA are as follows: mem -t 4 -k 32 -M -R. The mapping rate is between 98.64 to 98.93% for all strains. SAMTOOLS V1.9 (Li et al., 2009) was used to detect single nucleotide polymorphisms (SNPs) and small InDels (<50bp) with the following parameters: Mpileup -m 2 -F 0.002 -d 10000 -u -L 10000, and call --ploidy 1 -mv -Ov. The detected SNPs are further filtered with QUAL>20, as suggested by SAMTOOLS. Therefore, the final SNP list contains high-quality SNPs with high confidence. Subsequently, Integrative Genomics Viewer (IGV) was used to view the aligned sequence and perform further analysis on the identified SNPs/InDels (e.g., determination of amino acid substitution).

To verify our results, Snippy V4.6.0 (Seemann, 2015), a rapid haploid variant calling and core genome alignment software that has a built-in filter to detect high-quality SNPs, was used to reanalyze the whole-genome sequencing data. No difference was found in the identification of SNPs using SAMTOOLS and Snippy. Detailed information on all identified mutations in the evolved strains including the quality, read depth, and coverage of the SNPs, is available in Table S2.

BreakDancer V1.4.5 (Chen et al., 2009) was used to detect structural variations (SV) such as InDels larger than 50 bp, inversion, and translocation of large segments in the genome level, with the following parameters: breakdancer-max -s 50 -m 10000 -r 5-d Prefix. There are no large InDels or other structural variations detected in all of the evolved strains.

#### Quantification of gene expression level from RT-qPCR

For RT-qPCR data analysis, triplicates were applied for each gene, and the relative gene expression level was calculated based on the 2^−ΔΔCt^ method (Livak and Schmittgen, 2001)

#### Sequence database searching of proteomics data

The raw data were converted to mgf files by Bruker Compass DataAnalysis (version 5.2), and subsequently converted to mzML files by msconvert of the ProteoWizard (Kessner et al., 2008) (version 3.0.20229 64-bit). The mzML files were searched using Comet (version 2016.01 rev.2) (Eng et al., 2013) with a custom database. Briefly, the genome sequence of *S*. *aureus* ATCC 43300 was converted into a protein database using GeneMark (Lukashin and Borodovsky, 1998) (version 3.25) gene prediction tool. The proteins were then annotated using BLASTp (version 2.7.1) from NCBI using *S*. *aureus* NCTC 8325 as the protein database. The sequences of common contaminants, such as trypsin and human keratins, and decoy sequences generated by shuffling amino acid sequences between tryptic cleavage sites were added to the database. The decoy sequences in the database are used for the false discovery rate (FDR) estimation of the identified peptides. The search parameters criteria were set as follows: 15 ppm peptide mass tolerance, monoisotopic mass type, fully digested enzyme termini, 0.05 amu fragment bin tolerance, 0 amu fragment bin offset, carbamidomethylated cysteine, and oxidated methionine as the fixed and variable modifications respectively. The search results from Comet were processed by PeptideProphet (Keller et al., 2002), iProphet, and ProteinProphet of the Trans-Proteomics Pipeline (TPP) (Deutsch et al., 2010) in the decoy-assisted non-parametric mode. Every mzML run was analyzed independently. Protein identifications were filtered at a false discovery rate of 0.01 as predicted by ProteinProphet.

#### Label-free quantification of proteomics data by spectral counting

The proteins identified in at least two out of three biological replicates were used for label-free quantification by spectral counting. The quantification of proteins was given by the normalized spectral abundance factor (NSAF) (Paoletti et al., 2006), where the number of peptide-spectrum matches (PSMs) for each protein divided by the length of the corresponding protein is normalized to the total number of PSMs divided by the lengths of protein for all identified proteins. The differentially expressed proteins were filtered by the following cutoff: average spectral counts of at least three, the p-value for Student’s *t-*test on the NSAF values were lower than 0.05, and the fold changes were higher or lower than ± 1.5-folds.

#### Bioinformatics analysis

To determine if the sample preparation method used in this study offers comprehensive proteome profiling of *S*. *aureus* cells, we used PSORTb version 3.0.2 to predict protein localization (Yu et al., 2010). We visualize our proteomic data using principal component analysis (PCA) of the log NSAF values using the PCA function from the sklearn package with centering and scaling in python. We added 95% confidence intervals by calculating correlation matrices for the three replicates of each sample and then adding these intervals to our plot using the matplotlib package in python. To compare the protein expression profiles between different populations, we generated a heat map of fold changes of the differentially expressed proteins identified across the ancestral and evolved strains using the code developed in our lab. To find homologs of the hypothetical/unannotated proteins from the *S*. *aureus* NCTC8325 database obtained from UniProt (which we used in our database searching), we performed BLASTp homology search by NCBI to obtain protein homologs in other *S*. *aureus* strains that have sequence similarity above 99%. The list of protein homologs, along with the NCBI accession ID and the percent similarity was listed on the most right column in the Tables S3, S4, and S5. To highlight potentially important proteins among the differentially expressed proteins, STRING version 11.0 (Szklarczyk et al., 2016) was used to predict the protein-protein interactions and to visualize the interactions. DAVID (Database for Annotation, Visualization and Integrated Discovery) version 6.8 (Sherman and Lempicki, 2009) was used for gene ontology (GO) and pathway analysis.

## Data Availability

- Whole genome sequence data have been deposited in the BioProject database under the accession number PRJNA660918. The mass spectrometry proteomics data have been deposited to ProteomeXchange via the PRIDE repository with the dataset identifier PXD021667. - This paper does not report original code. - Any additional information required to reanalyze the data reported in this paper is available from the lead contact upon request.

## Supplemental information

Document S1. Figures S1–S6 and Tables S1, S2, and S6

Table S3. Differentially expressed proteins in the DAP-treated ancestral compared to untreated ancestral strain, DAP-treated S1D14 compared to untreated S1D14, DAP-treated S2D14 compared to the untreated S2D14, and DAP-treated S3D14 compared to the untreated S3D14, related to Figure 3

Table S4. Differentially expressed proteins in the S1D14 compared to ancestral, S2D14 compared to ancestral, and S3D14 compared to the ancestral, Related to Figure 4

Table S5. Differentially expressed proteins in the DAP-treated S1D14 compared to DAP-treated ancestral, DAP-treated S2D14 compared to DAP-treated ancestral, and DAP-treated S3D14 compared to DAP-treated ancestral, Related to Figure 4
