# Supplementary material for: Comparative proteomic investigation of multiple methicillin-resistant Staphylococcus aureus strains generated through adaptive laboratory evolution
Source: iScience. 2021 Aug 6;24(9):102950. doi: 10.1016/j.isci.2021.102950 (PMC8377494; doi:10.1016/j.isci.2021.102950)
Supplement: Document S1. Figures S1–S6 and Tables S1, S2, and S6 [file mmc1.pdf]

iScience, Volume *24*

Supplemental information

Comparative proteomic investigation of multiple methicillin-resistant *Staphylococcus aureus* strains generated through adaptive laboratory evolution

Jordy Evan Sulaiman, Lexin Long, Long Wu, Pei-Yuan Qian, and Henry Lam

Supplementary Figures

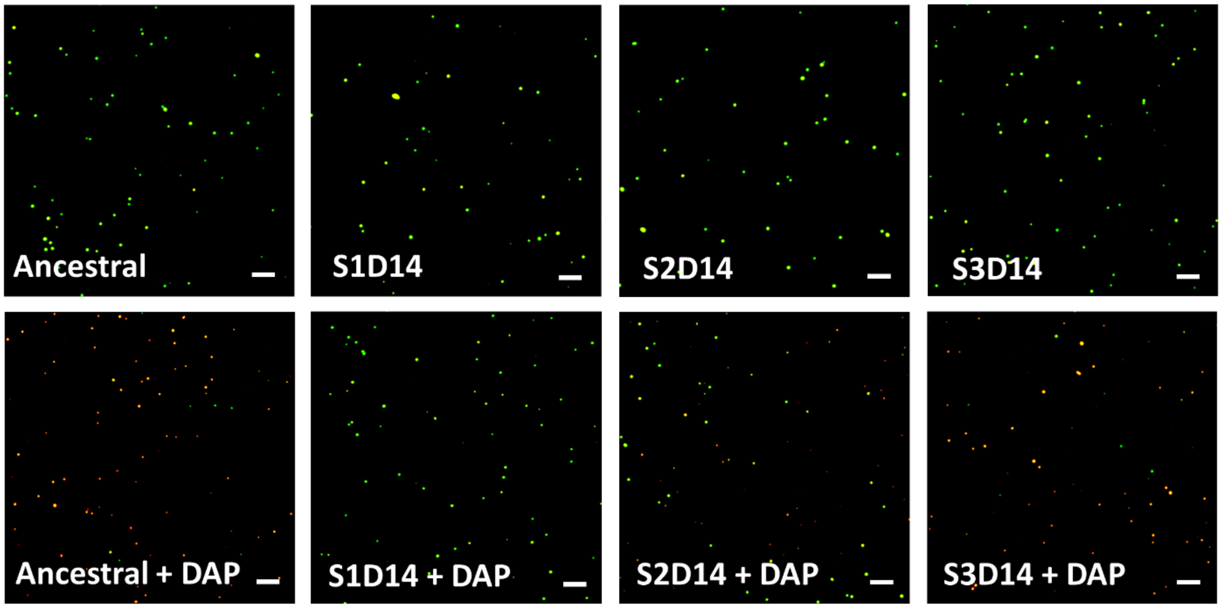

Figure S1. Epifluorescence microscopy of ancestral MRSA, S1D14, S2D14 and S3D14 before and after DAP treatment (10 µg/ml) for 1 h. Cells were stained with a LIVE/DEAD BacLight bacterial viability kit and visualized by epifluorescence microscopy. Green cells are viable cells and red cells are dead cells. The scale bars represent 50 µm. ***[Related to Figure 1]***

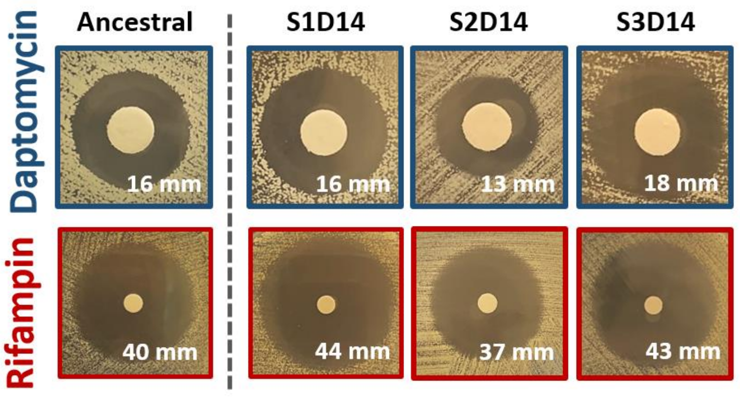

Figure S2. MIC test towards DAP and RIF carried out using disc diffusion antibiotic sensitivity testing. The text on the lower right corner marks the diameter of the zone of inhibitions. ***[Related to Figure 1]***

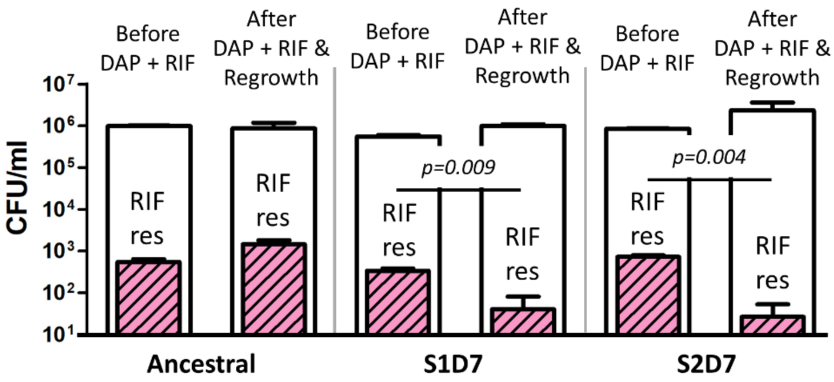

Figure S3. Competition experiments for RIF sensitive (empty bars) and resistant mutant (patterned fill). Around 10^3 RIF-resistant derivatives (*rpoB* H481Y) were mixed with 10^6 of their parental strains (ancestral, S1D7 or S2D7), treated with DAP (10 µg/ml) and RIF (1 µg/ml) combination for 1 h, and then regrown overnight. *P* values for the pairwise comparison were estimated with two-tailed Student's *t* test with unequal variances (mean ± s.e.m., n = 3). ***[Related to Figure 2]***

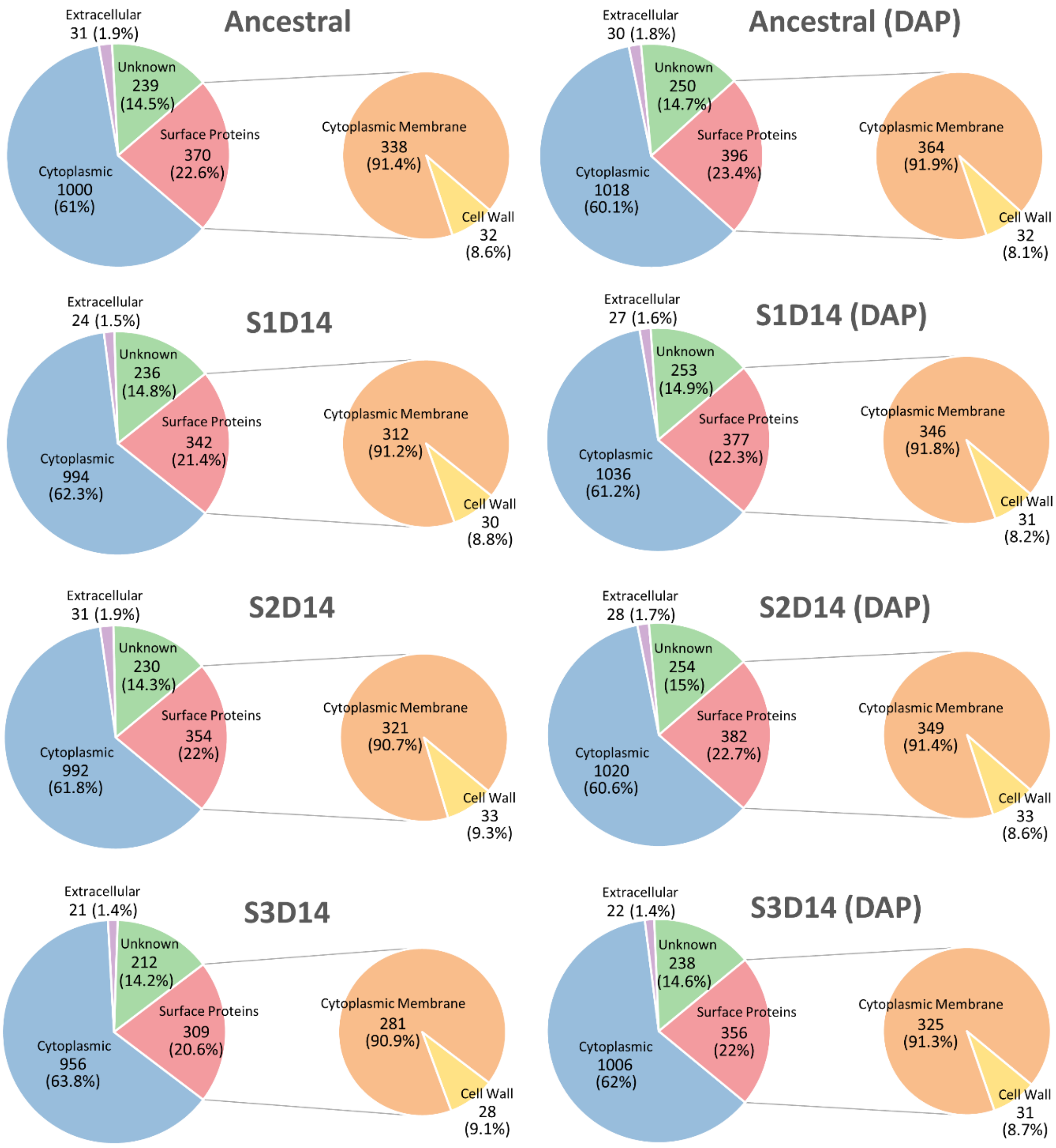

Figure S4. Localization distribution of the proteins identified in the untreated and DAP-treated ancestral, S1D14, S2D14 and S3D14 strains. Localization is predicted by PSORTb version 3.0.2. The identification of surface-associated proteins for each strain is ~350 proteins, which is more than 20% of the total identification. ***[Related to Figure 3]***

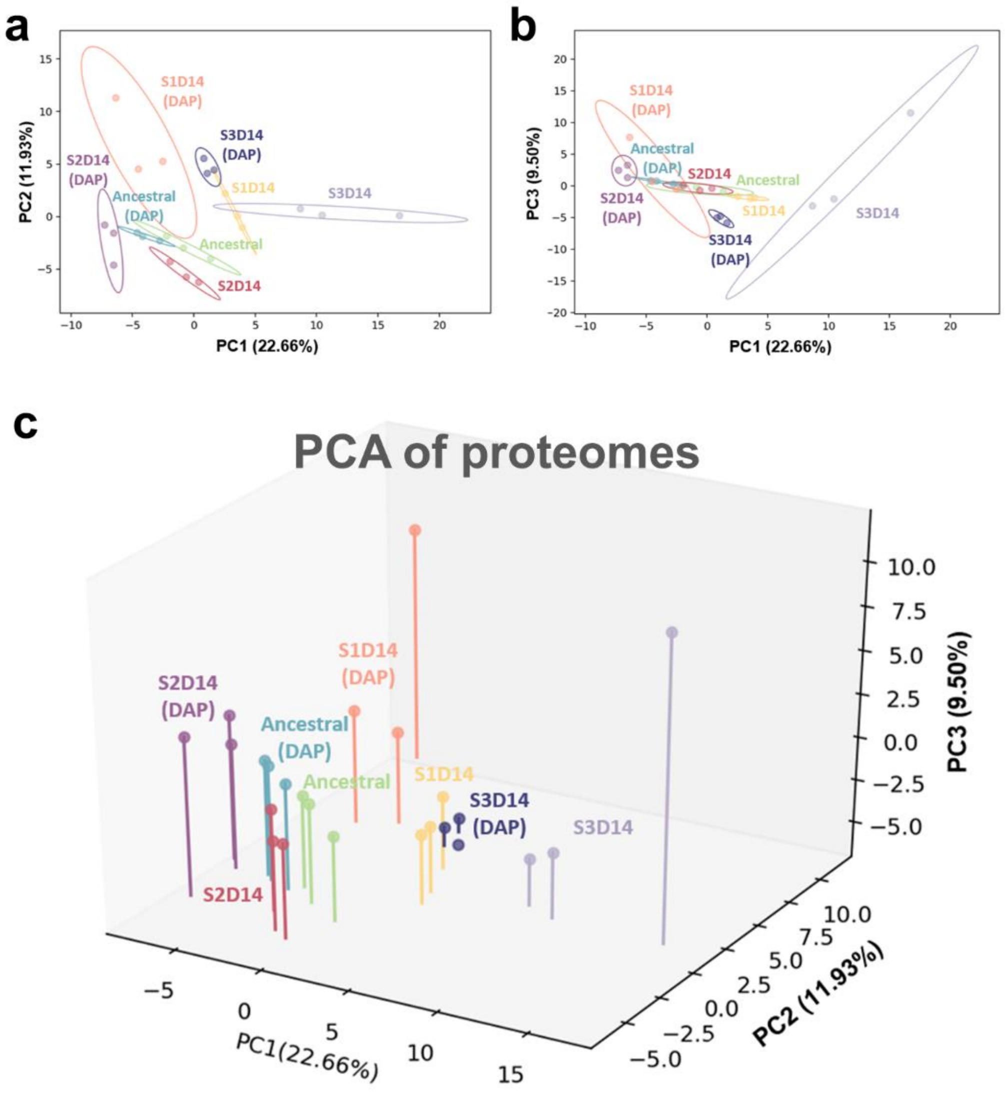

Figure S5. Principal Component Analysis (PCA) of proteomes. PC1 versus PC2 (**a**), PC1 versus PC3 (**b**), and PC1, PC2, and PC3 (**c**) are shown. Shaded circles represent 95% confidence intervals based on correlation matrices of the three replicates as follows: light green, ancestral; yellow, S1D14; red, S2D14; light purple, S3D14; dark green, ancestral after DAP treatment; orange, S1D14 after DAP treatment; dark purple, S2D14 after DAP treatment; dark blue, S3D14 after DAP treatment. ***[Related to Figure 3]***

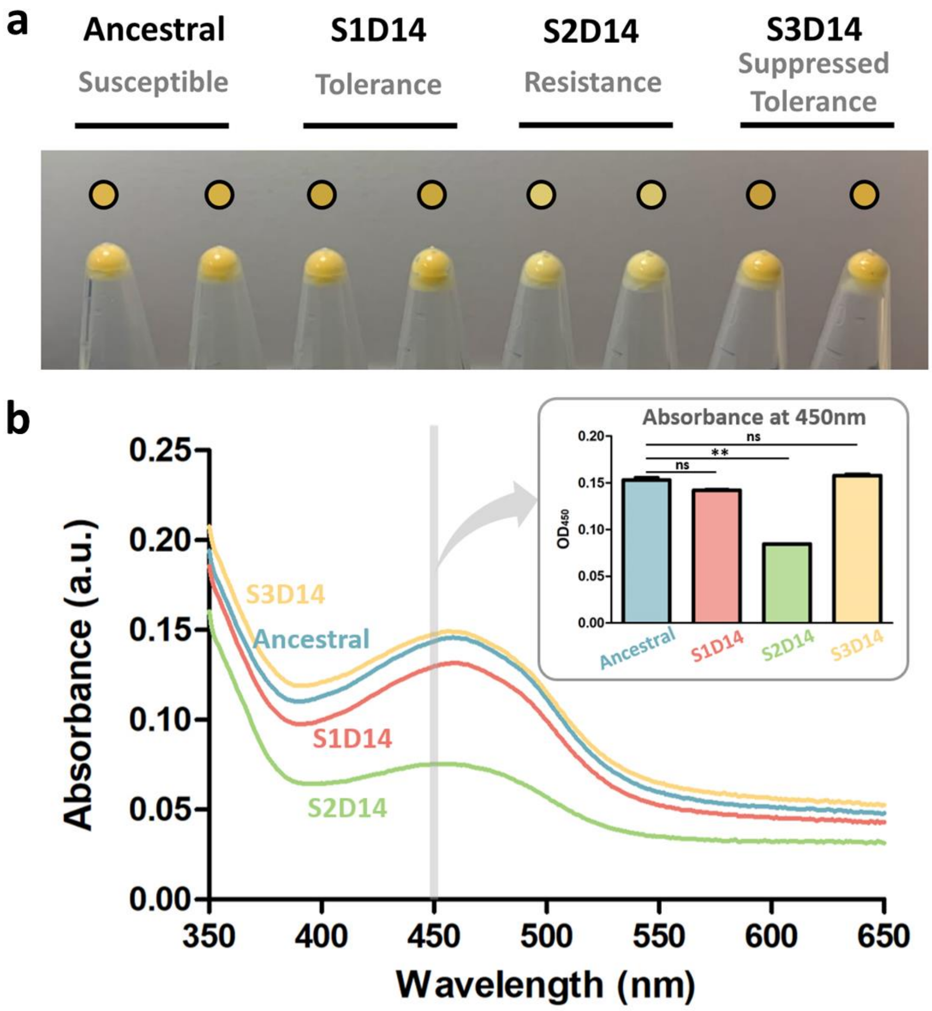

Figure S6. **Pigment quantification of the ancestral and the evolved strains. a,** Visualization of the pigment color of ancestral, S1D14, S2D14 and S3D14 strains after growth for 24 h. For each strain, cell pellets from two replicates are shown. The circle above the tube shows the color of the cell pellet. **b,** Absorbance spectrum of the carotenoids extracted from ancestral, S1D14, S2D14 and S3D14 through methanol extraction method. The inset figure shows another set of measurement of the absorbance (OD_{450}) of the carotenoids (mean ± s.e.m., n = 3). Significance of difference with the ancestral: ns, not significant, ** $P < 0.01$, (two-tailed t-test with unequal variances). ***[Related to Figure 4]***

Supplementary Tables

Table S1. Mutations detected from whole genome sequencing. *[Related to Figure 1 & 2]*

Contig no.	Gene	Position/ Mutation	Protein change	Annotation	Mutation presence							
					Ancestral-RIF	S1D7	S1 D7-RIF	S1 D14	S2D7	S2 D7-RIF	S2 D14	S3 D14
1	*mprF*	690886 / T > C	T345A	Phosphatidylglycerol lysyltransferase	-	-	-	-	+	+	+	-
1	Non-coding	783756 / C > A	-	9 bp upstream *pgsA* (CDP-diacylglycerol--glycerol-3-phosphate 3-phosphatidyltransferase)	-	+	+	+	-	-	-	+
1	*pta*	1451905 / G > A	L178F	Phosphate acetyltransferase	-	-	-	-	-	-	-	+
1	*rpoB*	1509675 / G > A	H481Y	DNA-directed RNA polymerase subunit beta	+	-	+	-	-	+	-	-
2	hp	5155 / C -> T	A290T	Hypothetical protein	-	-	-	-	-	-	-	+
DAP susceptibility					SUS	TOL	TOL	TOL	RES	RES	RES	Supp-ressed TOL
RIF susceptibility					RES	SUS	RES	SUS	SUS	RES	SUS	SUS

*Information about the database used for whole-genome sequencing:
File name: Staphylococcus_aureus_ATCC_43300.fasta (downloaded September 2020 from ATCC website), Assembly ID: 7e24d142adc2427d, Genome ID: 79691302ed634fef, Number of contig: 2 (circular). See **Table S2** for detailed information of these SN

Table S2. Detailed information on all identified mutations in the evolved strains from the whole-genome sequencing data, including the quality, read depth and coverage. ***[Related to Figure 1 & 2, and Table S1]***

Software/ Tool	Strain	Contig	Position	REF	ALT	Average Quality	Raw read depth	No. of high-quality ALT reads	No. of high-quality REF reads
**SAMTOOLS**	**Ancestral -RIF**	1	1509675	G	A	228	359	304	1
	**S1D7**	1	783756	C	A	225	355	314	0
	**S1D7-RIF**	1	783756	C	A	228	373	342	2
		1	1509675	G	A	228	423	373	1
	**S1D14**	1	783756	C	A	228	378	342	1
	**S2D7**	1	690886	T	C	225	361	324	0
	**S2D7-RIF**	1	690886	T	C	225	374	350	0
		1	1509675	G	A	225	476	418	0
	**S2D14**	1	690886	T	C	228	317	291	1
	**S3D14**	1	783756	C	A	225	380	329	0
		2	5155	C	T	228	502	431	5

	Strain	Contig	Position	REF	ALT	Total summed Quality	Total read depth at the locus	Count of full observations of the ALT haplotype	Sum of quality of the ALT observations	Count of full observations of the REF haplotype	Sum of quality of the REF observations
SNIPPY	**Ancestral-RIF**	1	1509675	G	A	10543.1	299	298	11853	1	41
	**S1D7**	1	783756	C	A	10702.2	303	302	11961	0	0
	**S1D7-RIF**	1	783756	C	A	10462.1	298	296	11748	2	82
		1	1509675	G	A	12247.5	353	352	13772	1	32
	**S1D14**	1	783756	C	A	10704.1	304	302	11974	1	22
	**S2D7**	1	690886	T	C	10812.6	305	305	12076	0	0
	**S2D7-RIF**	1	690886	T	C	10884.7	309	309	12165	0	0
		1	1509675	G	A	13648.2	393	392	15354	0	0
	**S2D14**	1	690886	T	C	9433.08	271	270	10579	1	41
	**S3D14**	1	783756	C	A	10461.6	296	296	11690	0	0
		2	5155	C	T	12980	367	366	14566	1	41

Table S6. Primers used for real-time PCR analysis. ***[Related to Figure 6]***

Gene	Forward Primer	Reverse Primer	Design
pgsA	TGGCTTCCCTTAGCGATTTTGT	CAGTTACGGCAAATTCTCTGGC	This study
pta	TGCTTGTTTATGCTGGTAAAGC	GTTCTTGATACACCTGGTTTCG	McClary et al. (2017) *Appl. Environ. Microbiol. 83(17)*: e01052-17.
gyrB	CGTTAATTGAAGCAGGCTATGTG	TGGTGTTGGATTCAATTCAGATT	Chen et al. (2018) *Front. Microbiol. 9*:1086.
